# A Fluorescent Probe for Imaging and Treating S-Nitrosation Stress in OGD/R Cells

**DOI:** 10.3390/antiox14030311

**Published:** 2025-03-04

**Authors:** Hui Ye, Chen Zhang, Lerong Li, Cunrui Li, Jiayue Yu, Duorui Ji, Zhuangzhuang Liang, Jianbing Wu, Zhangjian Huang

**Affiliations:** 1 State Key Laboratory of Natural Medicines, China Pharmaceutical University, Nanjing 211198, China; huiye@stu.cpu.edu.cn (H.Y.); chenzhang98@stu.cpu.edu.cn (C.Z.); 3124075304@stu.cpu.edu.cn (L.L.); cunruili@stu.cpu.edu.cn (C.L.); 3124074309@stu.cpu.edu.cn (J.Y.); duoruiji@stu.cpu.edu.cn (D.J.); lzz@stu.cpu.edu.cn (Z.L.); 2 Xinjiang Key Laboratory of Biopharmaceuticals and Medical Devices, Key Laboratory of Active Components of Xinjiang Natural Medicine and Drug Release Technology, Engineering Research Center of Xinjiang and Central Asian Medicine Resources, School of Pharmacy, Xinjiang Medical University, Urumqi 830054, China

**Keywords:** S-nitrosation, nitric oxide, S-nitrosylation, theranostic, OGD/R, imaging, treatment

## Abstract

Protein S-nitrosation, a redox post-translational modification elicited by nitric oxide (NO), is essential for modulating diverse protein functions and signaling pathways. Dysregulation of S-nitrosation is implicated in various pathological processes, including oxygen-glucose deprivation/reperfusion (OGD/R) injury, a widely used model for ischemia-reperfusion diseases. The dynamic changes in S-nitrosothiols (SNOs) during ischemia-reperfusion highlight the need for theranostic strategies to monitor and modulate SNO levels based on pathological progression. However, to date, no theranostic strategies have been reported for addressing dysregulated SNO in disease models, particularly in OGD/R conditions. Here, we report the development of a selective probe **P-EHC**, which could specifically react with SNOs to release **EHC**, not only exhibiting turn-on fluorescence with high quantum yield and good water solubility but also demonstrating macrophage migration inhibitory factor (MIF) inhibitory activity. In an OGD/R model of SH-SY5Y cells, we observed elevated SNO levels by using live-cell confocal imaging. Treatment of **P-EHC** significantly reduced intracellular reactive oxygen species (ROS), lowered total NO_x_ species, and improved cell viability in the OGD/R model. In summary, the simplicity and versatility of **P-EHC** suggest its broad applicability for monitoring SNO in various biological models and therapeutic contexts, particularly in ischemia-reperfusion diseases.

## 1. Introduction

Nitric oxide (NO) is a versatile gaseous signaling molecule with a wide range of physiological activities capable of inducing S-nitrosation modifications on protein cysteine residues [1,2]. Excessive levels of SNOs are closely associated with intracellular inflammatory responses and reflect the severity of ischemia-reperfusion (I/R) injury [3,4]. Elevated SNO concentrations exacerbate cellular damage by promoting inflammation and oxidative stress, contributing to tissue dysfunction [5]. Oxygen-glucose deprivation/reperfusion, a commonly used cell model for pressure ulcers and cerebral and myocardial ischemia, leads to increased SNO levels and cell death [6,7,8]. OGD/R triggers upregulation of inducible nitric oxide synthase (iNOS) expression [9] and neuronal nitric oxide synthase (nNOS) activity [10,11], leading to increased NO production. Moreover, under hypoxic conditions, inorganic nitrite can be converted to NO through enzymatic pathways (e.g., xanthine oxidase) [12] and non-enzymatic mechanisms (e.g., ascorbic acid-dependent reduction) [13,14]. NO can mediate protein S-nitrosation through auto-oxidation to dinitrogen trioxide (N_2_O_3_) [15] or oxidative nitrosation catalyzed by superoxide [16,17]. As a storage and bioactive form of NO [2], SNO levels increase under OGD/R conditions. Therefore, monitoring and regulating SNO levels is crucial for assessing the severity of ischemic injury and guiding therapeutic strategies [18]. Modulating excess SNOs presents a potential approach to mitigate inflammation, enhance tissue recovery, and improve treatment outcomes in ischemic conditions [19].

Fluorescent probes for SNOs have attracted considerable attention due to their potential for monitoring NO-related signaling pathways in biological systems. Early developments focused on phosphine-based probes [20,21,22,23] that selectively react with SNOs to produce a fluorescent signal, enabling the detection of SNO levels in cells. Various strategies have been employed, including the use of coumarin [24] and rhodamine derivatives [25], which provide high sensitivity and specificity for SNO detection. A fluorogenic probe has been applied to image the dynamic changes in protein S-nitrosation in live endothelial cells [26]. Recently, an indole-based fluorescent probe with unique long-wavelength emission was developed for the direct detection of SNOs in mitochondria [27]. While existing probes have made significant strides in detecting SNO levels in various biological systems, their application in disease models requiring both diagnostic and therapeutic functions remains a major challenge. However, no probe has been developed that not only enables precise monitoring of SNO dynamics in pathological models but also provides therapeutic benefits. Phosphine ester [28], which undergoes a rapid reductive ligation reaction targeting SNO moieties, has been utilized in the design of fluorescent probes for SNO detection [25,26,27]. When a phosphine ester reacts with SNO, the ester undergoes hydrolysis to yield an alcohol, along with one molecule of oxidized phosphine ester and 2-(diphenylphosphoryl)benzamide [28,29]. However, the poor water solubility of triphenylphosphine-based probes significantly limits their biological applications. Additionally, phosphine esters alone fail to provide effective cytoprotection in OGD/R models.

MIF is a multifunctional cytokine involved in various biological processes [30,31,32,33], including cellular responses to OGD/R injury [34]. MIF levels were elevated in plasma from patients with ischemic stroke and in the brains of focal ischemic rats [35]. Additionally, MIF expression was increased in hypoxia-treated human glioblastoma cells [36]. A recent study reported a ’pendular state’ of MIF, where 8 ng/mL of MIF promoted cell survival, while 50 ng/mL induced apoptosis in SH-SY5Y cells [37]. Under OGD/R conditions, MIF actively facilitated receptor-interacting protein kinase 1 (RIPK1)-mediated cell death [38]. Exogenous MIF administration increases infarct volume, whereas the MIF antagonist ISO-1 reduced infarct volume in tMCAo rats [39]. These studies suggest that regulating excessive MIF levels with MIF inhibitors holds promise as a therapeutic strategy in diseases characterized by pathological MIF upregulation.

**Figure 1:**
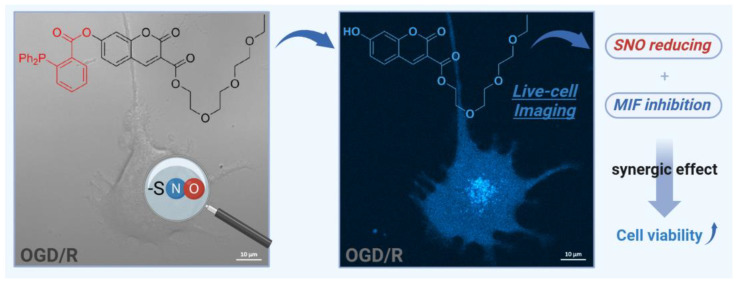
Design of **P-EHC**, a theranostic fluorescent probe for SNOs.

Herein, we present the design of a theranostic fluorescent probe for SNOs, **P-EHC** (Figure 1), which integrates an SNO-reactive moiety with the water-soluble fluorophore, **EHC**. Upon reaction with SNOs, **P-EHC** releases **EHC**, exposing a hydroxyl group and generating a strong fluorescence signal. Notably, **EHC** exhibited MIF inhibitory activity, which, in combination with the SNO-scavenging capability of the phosphine ester, synergistically protects cells under OGD/R conditions. Using **P-EHC**, we revealed the upregulation of SNO levels in OGD/R-treated SH-SY5Y cells. Additionally, **P-EHC** reduced intracellular ROS and total NO_x_ species in OGD/R cells. Collectively, these results highlight **P-EHC** as a powerful tool for SNO-based theranostic applications.

## 2. Materials and Methods

### 2.1. Information on General Chemistry and Reagents 

^1^H NMR and ^13^C NMR spectra were recorded with a Bruker Avance spectrometer at 303 K using TMS as an internal standard (Bruker, Billerica, MA, USA). MS spectra were recorded on a Mariner Mass Spectrum (ESI) and high-resolution mass spectrometry (HRMS) on an Agilent 1260-6230 TOF LC/MS (Santa Clara, CA, USA). Analytical TLC (HPTLC Silica Gel 60 GF 254, 2.5 × 5.0 cm) was bought from Leyan Co., Ltd. (Shanghai, China), and the chromatograms were visualized under UV light at 254 and 365 nm or under various colorants. All reagents used in the chemical synthesis were obtained from Leyan Co., Ltd., unless otherwise specified. The purity of all target compounds was determined by HPLC (Agilent 1220 Infinity II) (Santa Clara, CA, USA), and the compounds with a purity of >95% were used for the following experiments. All solvents were reagent-grade and, when necessary, were purified and dried by standard methods. Solutions after reactions and extractions were concentrated using a rotary evaporator operating at a reduced pressure of ca. 20 Torr. N-Nitro-L-arginine methyl ester hydrochloride (L-NAME; Abcam, Cambridge, UK), DETA NONOate (TargetMol, Boston, MA, USA), and spermine NONOate (MCE, Shanghai, China) were commercially purchased from their respective manufacturers.

### 2.2. Synthesis of Compound ***1***

In a 25 mL round-bottom flask, malonic acid (104 mg, 1 mmol, 1.0 equiv), EDCI (767 mg, 4 mmol, 4.0 equiv), DMAP (6.1 mg, 0.05 mmol, 0.05 equiv), and 2 mL anhydrous dichloromethane (DCM) were added. The reaction solution was stirred at r.t. for 30 min. Then, 2-(2-(2-ethoxyethoxy)ethoxy)ethanol (356 mg, 2 mmol, 2.0 equiv) was introduced into the flask. The air in the reaction vessel was replaced with nitrogen, and the mixture was stirred at room temperature for 8 h. The reaction progress was monitored by TLC, and upon completion, the reaction mixture was purified by column chromatography (PE/EA = 1:1) to yield a colorless oily liquid (369 mg, 87% yield). ^1^H NMR (Figure S1) (300 MHz, Chloroform-*d*) δ 4.36–4.01 (m, 4H), 3.71 (t, *J* = 4.6 Hz, 4H), 3.69–3.61 (m, 14H), 3.60–3.50 (m, 6H), 3.41 (s, 2H), 1.20 (td, *J* = 7.0, 3.6 Hz, 6H). ^13^C NMR (75 MHz, Chloroform-*d*) δ 168.40, 70.57, 70.50, 70.41, 70.31, 70.26, 69.59, 69.47, 68.70, 68.52, 66.62, 66.52, 64.50, 64.42, 61.26, 14.92, 14.72. HRMS (Figure S2) *m*/*z* calcd for C_19_H_37_O_10_, [M + H]^+^, 425.2381; found, 425.2378, ppm error −0.7.

### 2.3. Synthesis of ***EHC***

In a 25 mL round-bottom flask, 2,4-dihydroxybenzaldehyde (240.2 mg, 1.74 mmol, 1.0 equiv), compound **1** (738 mg, 1.74 mmol, 1.0 equiv), and 2 mL ethanol were added. Then, 0.1 mL piperidine was introduced into the flask, and the mixture was stirred at 60 °C for 2 h. The reaction’s progress was monitored by TLC. Upon completion, HCl (0.6 mL, 2 M in ethanol) was added to the reaction mixture. The reaction mixture was then purified by column chromatography (PE/EA = 1:2), yielding a light yellow solid (287 mg, 45% yield). ^1^H NMR (Figure S3) (300 MHz, Chloroform-*d*) δ 8.31 (s, 1H), 7.34 (d, *J* = 8.5 Hz, 1H), 6.85 (dd, *J* = 8.5, 2.3 Hz, 1H), 6.79 (d, *J* = 2.2 Hz, 1H), 4.48–4.39 (m, 2H), 3.90–3.81 (m, 2H), 3.74 (d, *J* = 5.5 Hz, 2H), 3.70 (d, *J* = 5.5 Hz, 2H), 3.65–3.61 (m, 2H), 3.55 (t, *J* = 4.6 Hz, 2H), 3.50–3.42 (m, 2H), 1.13 (t, *J* = 7.0 Hz, 3H). ^13^C NMR (75 MHz, CDCl_3_) δ 164.01, 163.15, 157.32, 149.34, 131.15, 114.86, 111.98, 110.76, 102.96, 77.51, 77.09, 76.66, 70.58, 70.52, 70.42, 69.60, 69.05, 66.61, 64.47, 14.97. HRMS (Figure S4) *m*/*z* calcd for C_18_H_22_O_8_, [M + Na]^+^, 389.1207; found, 389.1216, ppm error +2.3. HPLC purity: 97.01% (Figure S7).

### 2.4. Synthesis of ***P-EHC***

In a 25 mL round-bottom flask, 2-(diphenylphosphino) benzoic acid (239.7 mg, 0.78 mmol, 1.0 equiv), EDCI (299.5 mg, 1.56 mmol, 2.0 equiv), DMAP (4.8 mg, 0.039 mmol, 0.05 equiv), and 2 mL DCM were added. The reaction solution was stirred at r.t. for 30 min. **EHC** (287.0 mg, 0.78 mmol, 1.0 equiv) was then added. The reaction mixture was stirred at room temperature for 8 h. The reaction’s progress was monitored by TLC, and upon completion, the mixture was purified by column chromatography (PE/EA = 1:1) to yield a light yellow solid (372.5 mg, 71% yield). ^1^H NMR (Figure S5) (600 MHz, Chloroform-*d*) δ 8.51 (s, 1H), 8.24 (ddt, *J* = 7.6, 5.9, 4.1 Hz, 1H), 7.57–7.53 (m, 1H), 7.52–7.44 (m, 2H), 7.38–7.26 (m, 10H), 7.02 (ddd, *J* = 7.3, 3.9, 2.0 Hz, 1H), 6.96 (d, *J* = 7.5 Hz, 2H), 4.51–4.46 (m, 2H), 3.86–3.82 (m, 2H), 3.74–3.70 (m, 2H), 3.70–3.64 (m, 4H), 3.58 (dd, *J* = 5.9, 3.8 Hz, 2H), 3.51 (q, *J* = 7.0 Hz, 2H), 1.19 (t, *J* = 7.0 Hz, 3H). ^13^C NMR (151 MHz, CDCl_3_) δ 164.27, 162.68, 156.16, 155.90, 155.21, 148.20, 141.95, 141.76, 137.23, 137.15, 134.55, 134.10, 133.96, 133.06, 132.51, 132.39, 131.54, 131.53, 130.31, 129.01, 128.70, 128.65, 128.45, 119.05, 117.09, 115.58, 110.23, 77.35, 77.14, 76.92, 70.72, 70.69, 70.66, 69.82, 68.89, 66.60, 64.91, 15.17. HRMS (Figure S6) *m*/*z* calcd for C_39_H_34_O_9_P, [M + Na]^+^, 677.1911; found, 677.1925, ppm error +2.1. HPLC purity: 97.50% (Figure S8).

### 2.5. Docking Studies

The conformation of **EHC** was optimized with Chem 3D 17.0. Molecular docking and visualization were performed using Schrödinger 2009. The MIF structure used was crystallographically determined and retrieved from the Protein Databank (ID: 1GCZ).

### 2.6. General Procedure for UV Absorbance and Fluorescence Detection

The UV absorption scan of **EHC** was performed in methanol, with the methanol absorbance subtracted as the background. The fluorescence properties of **EHC** were measured in PBS containing 5% DMSO (pH 7.4, unless otherwise specified), except for pH-dependent experiments. Unless otherwise stated, **P-EHC** was dissolved in PBS containing 5% DMSO and mixed with an equal volume of reactant. The mixture was shaken at room temperature, and signals were detected using an EnSpire Multilabel Reader (PerkinElmer, Waltham, MA, USA).

### 2.7. MIF Tautomerase Activity Assay

For the detection of MIF activity in biological samples, a reported tautomerase assay was employed with modifications [40]. SH-SY5Y cells were cultured with standard growth medium in T25 flasks until 80% confluence. Cells were lysed using lysis buffer (Beyotime, P0013) (Shanghai, China) containing 1 mM PMSF (Adamas life, E8060) (Shanghai, China). Then, 4 mM L-3,4-dihydroxyphenylalanine methyl ester hydrochloride (Leyan) (Shanghai, China) and 20 mM sodium m-periodate (Sigma-Aldrich) (St. Louis, MO, USA) were freshly prepared in water. After, 20 μL of the lysate in 10 mM sodium phosphate/1 mM EDTA (pH 6.2) was pipetted into a 96-well ELISA plate. Then, 1.1 μL of the test compounds (in DMSO, with a final concentration of 10 μM) were added to the plate. Two hundred microliters of the substrate (L-dopachrome methyl ester), prepared by mixing 72 μL of sodium m-periodate stock with 108 μL of L-3,4-dihydroxyphenylalanine methyl ester hydrochloride in 1620 μL of sodium phosphate/EDTA (pH 6.2), were added. The samples were mixed and the decrease in Abs475 due to dopachrome tautomerization was monitored for 300 s using Berthold Tristar5 (Berthold, Bad Wildbad, Germany).

### 2.8. Partition Coefficient (LogP)

The LogP value was determined using the shake-flask method as the logarithm of the ratio of the compound’s concentration in the n-octanol phase to that in the aqueous phase. Since the compound’s concentration (C) is proportional to its absorbance in solution, absorbance measurements were used to calculate the partition coefficient (P). Briefly, **EHC** was dissolved in PBS (1×, pH 7.4), and its absorbance at 402 nm (denoted as Abs_PBS_) was measured using a plate reader (Berthold Tristar5). N-octanol was then added, and the mixture was vigorously shaken to ensure equilibrium. The absorbance in the PBS phase after shaking (Abs_residue_) was subsequently measured. To account for the molar absorptivity difference, the absorbance in the n-octanol phase was calculated by subtracting Abs_residue_ from Abs_PBS_. The LogP value was calculated using the following equation:

(1) $$\mathrm{LogP}=\mathrm{Log}\;\frac{C_{oil}}{C_{PBS}}=\mathrm{Log}\;\frac{{Abs}_{PBS}-{Abs}_{residue}}{{Abs}_{PBS}}$$

### 2.9. Fluorescence Quantum Yield

Relative quantum yields were measured in PBS containing 5% DMSO and at 25 °C by a relative method using optically matching solutions of 9,10-diphenylanthracene (DPHA) (Φ*_f_* = 0.92 in cyclohexane) as a standard at an excitation wavelength of 330 nm [41]. The following equation was used to determine the relative fluorescence quantum yield:

(2) $$\Phi_{s}=\Phi_{f}\cdot \frac{A_{r}\cdot \sum F_{s}\cdot n_{s}^{2}}{A_{s}\cdot \sum F_{r}\cdot n_{r}^{2}}$$

where *A* (*A*_s_ = 0.049, *A*_r_ = 0.031) are the absorbance of the sample and the reference, respectively, at the same excitation wavelength, ∑F ($\sum F_{s}$ = 7236851, $\sum F_{r}$ = 4366436) are the corresponding relative integrated fluorescence intensities, and *n* (*n*_s_ = 1.333; *n*_r_ = 1.426) is the refractive index of the solvent.

### 2.10. LC-MS Analysis

Equal volumes of **P-EHC** (6 mM in MeOH) and S-nitrosoglutathione (GSNO) (3 mM in 1× PBS) were mixed and stirred at room temperature under N_2_ protection for 2 h. The reaction mixture was injected into a Waters analytical system and analyzed using a mobile phase (methanol/water = 65:35) at a flow rate of 0.3 mL/min. UV absorption of the compounds was monitored at 254 nm and molecular weights were determined by ESI mass spectrometry.

### 2.11. Cell Culture and OGD/R Modeling

SH-SY5Y cells were purchased from iCell Bioscience Inc. (Shanghai, China). SH-SY5Y cells were seeded and cultured at 37 °C with 5% CO_2_ for 24 h. After washing, the cells were cultured in glucose- and FBS-free DMEM medium (with or without test compounds) under hypoxic conditions (1% O_2_) at 37 °C for 16 h. The cells were then cultured in a complete DMEM medium at 37 °C with 95% air and 5% CO_2_ for 24 h.

### 2.12. Cell Culture in Glass-Bottom Dishes

Cells were digested, counted, and prepared as a suspension at a concentration of 8 × 10^4^ cells/mL. A 200 μL aliquot of the cell suspension was added to each glass-bottom cell culture dish (φ15 mm) (NEST, Wuxi, China) and incubated at 37 °C with 5% CO_2_ for 3 h. Subsequently, 1.3 mL of complete medium was added to each dish, and incubation continued at 37 °C with 5% CO_2_ for 24 h. These cells were then used for staining and confocal imaging.

### 2.13. SNO Staining in Living Cells

Two milliliters of complete medium (with or without NONOate) were added to each dish to replace the original culture medium, and the cells were incubated at 37 °C with 5% CO_2_ for 4 h. A DMSO stock solution of **P-EHC** was then added to the glass-bottom cell culture dishes to a final concentration of 10 μM, followed by additional incubation for 4 h. The culture medium was then carefully removed, and the dishes were gently washed with PBS. Finally, 200 μL of PBS was added before confocal microscopy observation.

### 2.14. Triple Staining for Dead Cells, Live Cells, and Intracellular SNOs

**P-EHC** staining solution (final concentration 10 μM) was added to the glass-bottom cell culture dishes and incubated for 8 h. For OGD/R-treated cells, **P-EHC** was added during the reperfusion phase, 16 h after OGD. Subsequently, Calcein AM staining solution (final concentration 2 μM) (Beyotime, Shanghai, China) was added to the culture medium and incubated for 15 min. Propidium iodide (PI, final concentration 50 μg/mL) (Biofroxx, Einhausen, Germany) was then added for a 5 min incubation period. After gently washing away the triple staining solution with PBS, the cells were observed using a laser confocal microscope.

### 2.15. Confocal Imaging and Fluorescence Intensity Quantification

Cell imaging was performed using a confocal microscope (ZEISS LSM800) (Oberkochen, Germany) with a 63× oil-immersion objective. The excitation wavelengths for each channel were as follows: blue channel, λ_ex_ = 405 nm; green channel, λ_ex_ = 488 nm; and red channel, λ_ex_ = 561 nm. Emission light was detected using the optimal signal mode. Fluorescence intensity was quantified through grayscale analysis using ZEN 2012 (version 1.1.2.0, blue edition) software, where the arithmetic mean intensity of each channel for individual cells was measured. Each data point represents the mean fluorescence intensity of viable cells from an independent experiment.

### 2.16. CCK-8 Assay

Each well of a 96-well plate was seeded with 200 μL of the cell suspension containing 8000 cells. After removing the culture medium, the wells were washed with PBS, followed by the addition of 180 μL of PBS per well. Subsequently, 20 μL of CCK-8 reagent (Beyotime) (Shanghai, China) was added to both OGD/R-treated and untreated plates. The plates were incubated at room temperature for 2 h, and the absorbance at 450 nm was measured to assess cell viability.

### 2.17. ROS Assay

Intracellular generation of ROS was detected using a DCFH-DA fluorescent probe according to the manufacturer’s instructions (Adamas life, C8093) (Shanghai, China). Briefly, SH-SY5Y cells were digested, counted, and prepared as a suspension at a concentration of 1 × 10^6^ cells/mL. The suspension was incubated with DCFH-DA (10 µM) diluted in DMEM in a humidified atmosphere of 5% CO_2_ (37 °C, 0.5 h). After being washed a third time with the same buffer without DCFH-DA, the cells were resuspended and measured using a plate reader (Berthold Tristar5, λ_ex_ = 488 nm/λ_em_ = 525 nm).

### 2.18. Total NO_x_ Species Assay

Total NO_x_ species (including NO, nitrite, and nitrate) were determined using the Total Nitric Oxide Assay Kit (Beyotime, S0023) (Shanghai, China) according to the manufacturer’s instructions. With the improved Griess reaction method, the total NO_x_ species were determined by measuring the concentrations of endogenous nitrite, nitrite converted from nitrate, and nitrite converted from NO. Briefly, SH-SY5Y cells were collected and lysed using lysis buffer for the nitric oxide assay (Beyotime, S3090) (Shanghai, China). NADPH, FAD, and nitrate reductase were then added to the cell lysates and incubated at 37 °C for 30 min to reduce nitrate to nitrite. Subsequently, LDH buffer and LDH were added, followed by incubation at 37 °C for an additional 30 min. After adding Griess Reagent 1, immediately followed by Griess Reagent 2, the lysates were incubated at 25 °C for 10 min. The absorbance at 540 nm was measured using a microplate reader (Berthold Tristar5). A nitrite standard curve was drawn for each experiment to calculate NO concentrations.

### 2.19. Statistical Evaluation

Statistical analysis was performed using Graphpad Prism 7.0 software. Unless otherwise stated, all values are expressed as the mean ± SD of n determinations or as mentioned. A *p*-value less than 0.05 was considered significant.

## 3. Results

### 3.1. Design, Synthesis, and Evaluation of ***P-EHC***

#### 3.1.1. **EHC**, a Coumarin Derivative with MIF Inhibitory Activity

**Figure 2:**
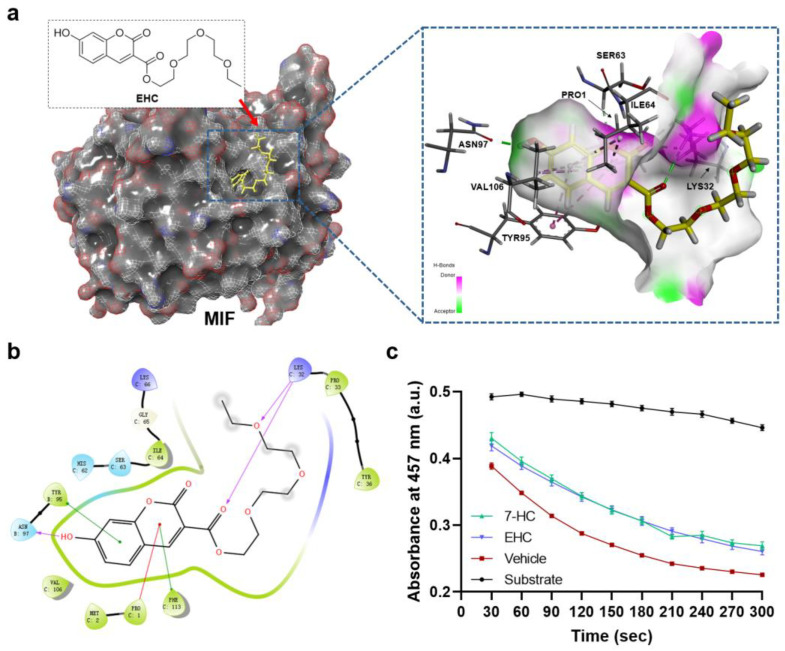
The design of **EHC**, a coumarin derivative with MIF inhibitory activity. (**a**) Chemical Structure of **EHC** and its 3D binding mode with MIF. The 3D ball-and-stick model of **EHC** is highlighted in gold. Localized 3D interaction model highlighting hydrogen bonds: donors are in magenta and acceptors are in green (PDB ID of MIF: 1GCZ). Interactions are represented by dashed lines, and arrows indicate amino acid residues. (**b**) A 2D interaction diagram of **EHC** with MIF. Purple arrow, H-bond; Green line, π–π stacking; Red line, π–cation interaction. (**c**) Inhibition of MIF-catalyzed dopachrome tautomerization by the vehicle, **7-HC**, and **EHC** (10 μM in 0.5% DMSO). The substrate, an L-dopachrome methyl ester, was monitored at 457 nm. Data are presented as the mean ± SD (*n* = 3).

7-Hydroxycoumarin (**7-HC**), a “turn-on” fluorescent scaffold [42] with MIF inhibitory activity [43,44,45], exhibits limited water solubility, presenting challenges for developing SNO probes suitable for biological applications. Guided by the co-crystal structures of coumarin derivatives bound to MIF (PDB ID: 1GCZ) [44], we designed and synthesized **EHC**, a **7-HC** derivative bearing a 2-(2-(2-ethoxyethoxy)ethoxy)ethyl substituent. **EHC** demonstrated improved solubility in PBS (1×, pH 7.4) at 2.52 mg/mL and a partition coefficient (LogP) of 0.33. Molecular docking analysis indicated that **EHC** occupies a binding cavity on MIF (Figure 2a) similar to that of **7-HC** and exhibits comparable interactions with MIF [44]. The 2-(2-(2-ethoxyethoxy)ethoxy)ethyl substituent is positioned in a solvent-exposed region (Figure 2a). The 2D interaction diagram of **EHC** with MIF highlights critical interactions, including π–π stacking with Tyr95 and Phe113, a π–cation interaction with Pro1, and hydrogen bonding with Asn97 and Lys32 (Figure 2b). In a tautomerase inhibition assay, **EHC** demonstrated comparable MIF inhibitory activity to **7-HC** in vitro (Figure 2c), supporting its potential as a therapeutic candidate for MIF-mediated OGD/R injury.

#### 3.1.2. Characterization of the Fluorescent Properties of **EHC**

**Figure 3:**
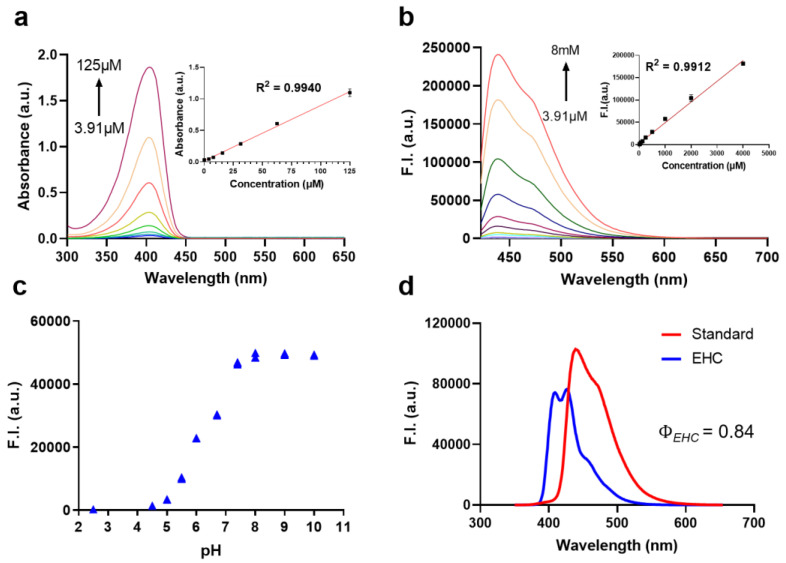
Characterization of the fluorescent properties of **EHC**. (**a**) UV–vis absorbance spectra of **EHC**. Inset: Plot of Abs402 against **EHC** concentrations (3.91−125 μM). (**b**) Fluorescence emission spectrum of **EHC** (λ_ex_ = 402 nm). Inset: Linear plot of fluorescence intensity at 438 nm versus **EHC** concentrations (3.91 μM–8 mM). For (**a**,**b**), different colored lines correspond to spectra at varying concentrations. (**c**) Fluorescence spectra of **EHC** in buffer with different pH levels (λ_ex_ = 402 nm, λ_em_ = 438 nm). (**d**) Fluorescence emission spectra of a standard compound (**DPHA**) in cyclohexane and **EHC** in PBS (1×, pH 7.4, containing 5% DMSO), both excited at 330 nm.

The UV–Vis spectrum of **EHC** shows maximum absorption at 402 nm (Figure 2a), with a strong linear relationship between absorbance and concentration in the range of 3.91 μM to 125 μM (R^2^ = 0.9940, Figure 3a inset). Upon excitation at 402 nm, **EHC** emitted fluorescence maximally at 438 nm, with fluorescence intensity linearly correlated to concentration from 3.91 μM to 8 mM (R^2^ = 0.9912, Figure 3b inset). Similar to **7-HC**, **EHC** displayed pH-sensitive fluorescence, with reduced intensity under acidic conditions (Figure 3c). Using DPHA as a standard, **EHC** achieved a high fluorescence quantum yield (ϕ***_EHC_*** = 0.84) (Figure 3d), surpassing that of **7-HC** (ϕ***_7-HC_***= 0.76) [46]. The introduction of the 2-(2-(2-ethoxyethoxy)ethoxy)ethyl substituent significantly improved water solubility, enhanced fluorescence properties, and retained MIF-inhibitory activity, establishing **EHC** as a promising fluorophore for both therapeutic and imaging applications.

#### 3.1.3. Design, Synthesis, and LC-MS Analysis of **P-EHC**

**Scheme 1:**
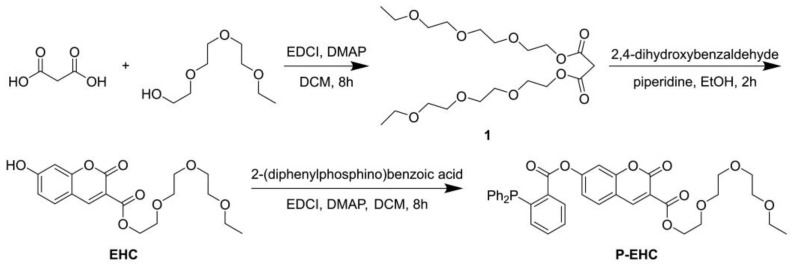
Synthetic route of **P-EHC**.

We condensed **EHC** with 2-(diphenylphosphino)benzoic acid to obtain **P-EHC** (Scheme 1).

**Figure 4:**
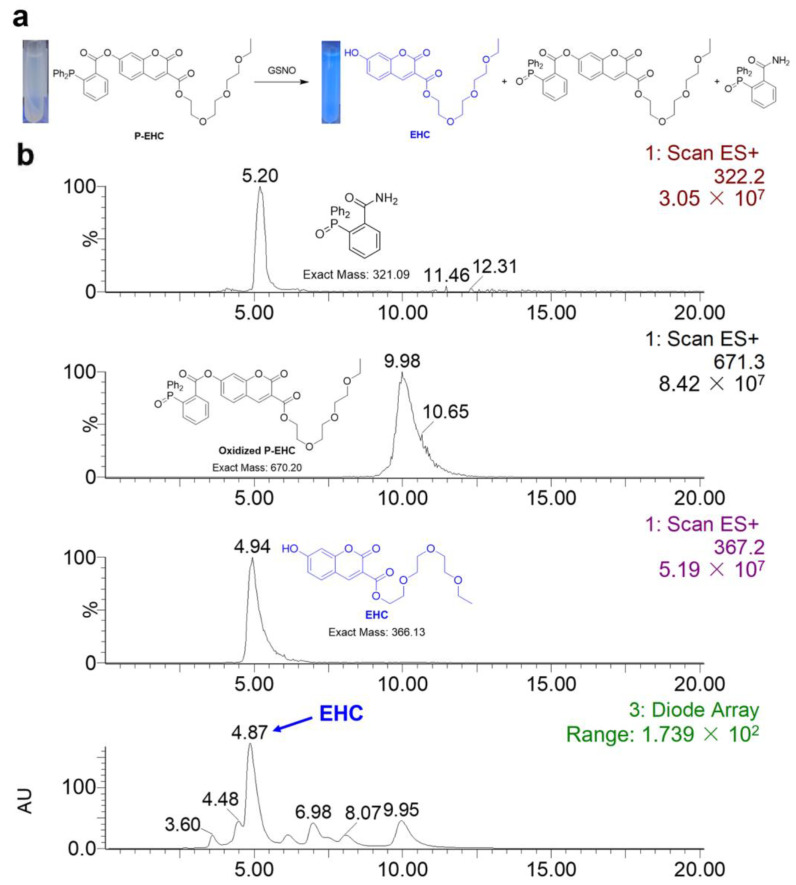
Reaction products **P-EHC** and GSNO. (**a**) Reaction scheme of **P-EHC** (3 mM) with GSNO (1.5 mM). The photographs of the reaction solution are overlaid on the equation. (**b**) LC-MS analysis of the reaction of **P-EHC** with GSNO after 2 h. The bottom panel shows the UV spectrum of the reaction, with the peak corresponding to **EHC** labeled. The panels above (from top to bottom) display the extracted ion chromatograms for 2-(diphenylphosphoryl)benzamide, **EHC**, and oxidized **P-EHC**. The structures and exact masses of the compounds are annotated on their respective spectra.

Upon reacting with GSNO, **P-EHC** released **EHC** and exhibited significant fluorescence under UV light (Figure 4a). To further confirm the detection mechanisms of **P-EHC**, we employed LC-MS to detect the reaction of **P-EHC** with GSNO. After a 2 h reaction, **EHC**, oxidized **P-EHC**, and 2-(diphenylphosphoryl)benzamide were detected in the reaction solution using LC-MS (Figure 4b). These products confirm that the reaction between **P-EHC** and GSNO follows a reductive ligation mechanism [28]. Notably, the strong MS signal of **EHC** further validates the ability of **P-EHC** to release the fluorescent **EHC** upon reaction with SNOs.

#### 3.1.4. In Vitro Reactivity Characterization of **P-EHC**

**Figure 5:**
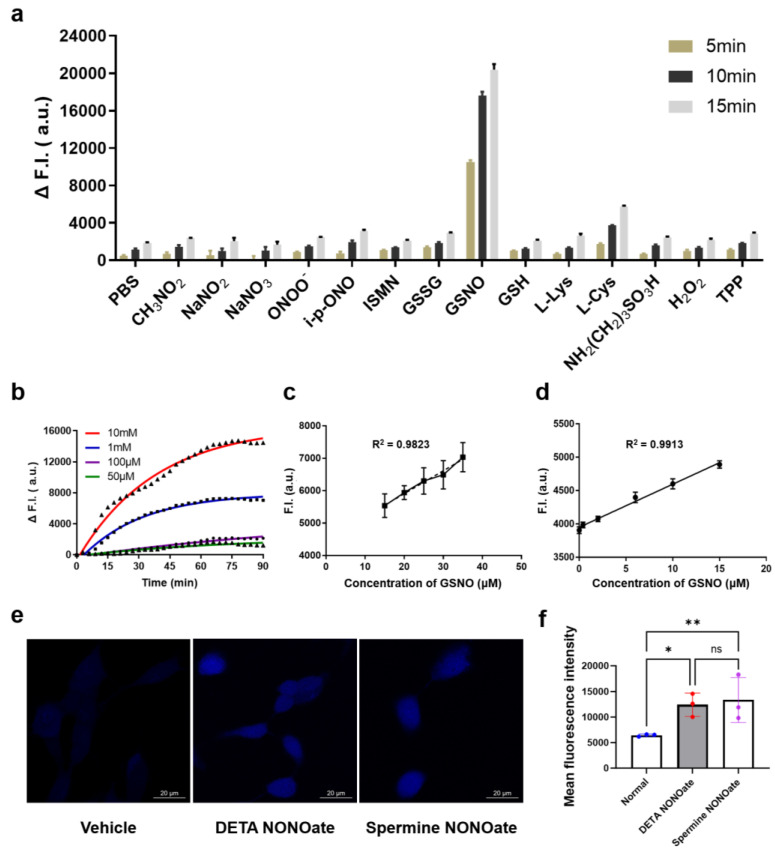
In vitro reactivity characterization of **P-EHC**. (**a**) Fluorescence enhancement of 100 μM **P-EHC** upon reacting with 10 mM of various compounds. i-p-ONO, isopropyl nitrite; ISMN, isosorbide mononitrate; TPP, triphenylphosphine. (**b**) Kinetic curves for the reaction of 100 μM **P-EHC** with varying concentrations of GSNO. (**c**) Standard curve of fluorescence intensity versus GSNO concentration for the reaction of 10 μM **P-EHC** with GSNO after 15 min. (**d**) Fluorescence response of 10 μM **P-EHC** to different GSNO concentrations in 20% fetal calf plasma dissolved in PBS. For (**a**–**d**), fluorescence intensity was determined by a plate reader. (**e**) Representative confocal micrographs of SH-SY5Y cells incubated with 10 μM **P-EHC** for 4 h, in the presence or absence of 10 μM DETA NONOate or spermine NONOate. λ_ex_ = 405 nm; Scale bar = 20 μm. (**f**) Statistical analysis of the experiment shown in (**e**). Each data point represents the mean fluorescence intensity of viable cells from an independent experiment, quantified by grayscale analysis. Analyzed by one-way ANOVA with Tukey’s multiple comparisons test. * *p* < 0.05, ** *p* < 0.01, ns = non-significant (*p* > 0.05). For (**a**–**d**,**f**), data are expressed as mean ± SD (*n* = 3).

Next, we evaluated the reaction selectivity of **P-EHC** (Figure 5a). In vitro fluorescence assays showed that **P-EHC** exhibited strong fluorescence upon reacting with GSNO. In contrast, minimal fluorescence enhancement was observed with 10 mM (100 eq) of various compounds, including nitromethane (CH_3_NO_2_), sodium nitrite (NaNO_2_), sodium nitrate (NaNO_3_), peroxynitrite (ONOO^−^), isopropyl nitrite (i-p-ONO), isosorbide mononitrate (ISMN), oxidized glutathione (GSSG), glutathione (GSH), L-lysine (L-Lys), L-cysteine (L-Cys), cysteine sulfonic acid (NH_2_(CH_2_)_3_SO_3_H), hydrogen peroxide (H_2_O_2_), and triphenylphosphine (TPP). A modest fluorescence enhancement was observed with L-Cys, which was significantly lower than that induced by equimolar GSNO. These results demonstrate that **P-EHC** possesses good reaction selectivity.

Kinetic studies showed that the reaction between **P-EHC** and GSNO reached a fluorescence plateau after approximately 75 min (Figure 5b). In PBS (1×, pH 7.4, containing 5% DMSO), the fluorescence response exhibited good linearity with GSNO concentrations in the range of 15–25 μM (R^2^ = 0.9823) (Figure 5c). Similarly, in 20% fetal bovine plasma, the reaction between **P-EHC** and varying concentrations of GSNO also demonstrated linearity (R^2^ = 0.9913) (Figure 5d).

After confirming the reactivity of **P-EHC** under cell-free conditions, we next investigated whether **P-EHC** could be used for SNO imaging in live cells. The incubation of SH-SY5Y cells with **P-EHC** resulted in weak fluorescence (Figure 5e), likely reflecting baseline intracellular SNO levels. In contrast, pretreatment of SH-SY5Y cells with DETA NONOate or spermine NONOate, commercially available NO donors that release NO through spontaneous dissociation under neutral or acidic conditions and are commonly used to induce S-nitrosation modifications [47,48], significantly increased the mean cellular fluorescence intensity compared to untreated cells (Figure 5f). In PBS solution, no significant change in fluorescence was observed when **P-EHC** was incubated with equimolar DETA NONOate and spermine NONOate for 15 min (Figure S9), indicating that **P-EHC** specifically reacts with SNOs formed from cysteine residues in cells rather than reacting directly with NO. Taken together, these findings demonstrate that **P-EHC** is a specific SNO probe capable of detecting SNOs at the cellular level, including both basal SNOs and SNOs induced by NO donors.

### 3.2. Theranostic Effects of ***P-EHC*** on OGD/R Cells

#### 3.2.1. Triple Staining for Dead Cells, Live Cells, and Intracellular SNOs in an OGD/R Model

Following the characterization of the probe’s reactivity, we investigated the theranostic effects of **P-EHC** on OGD/R-treated cells.

**Figure 6:**
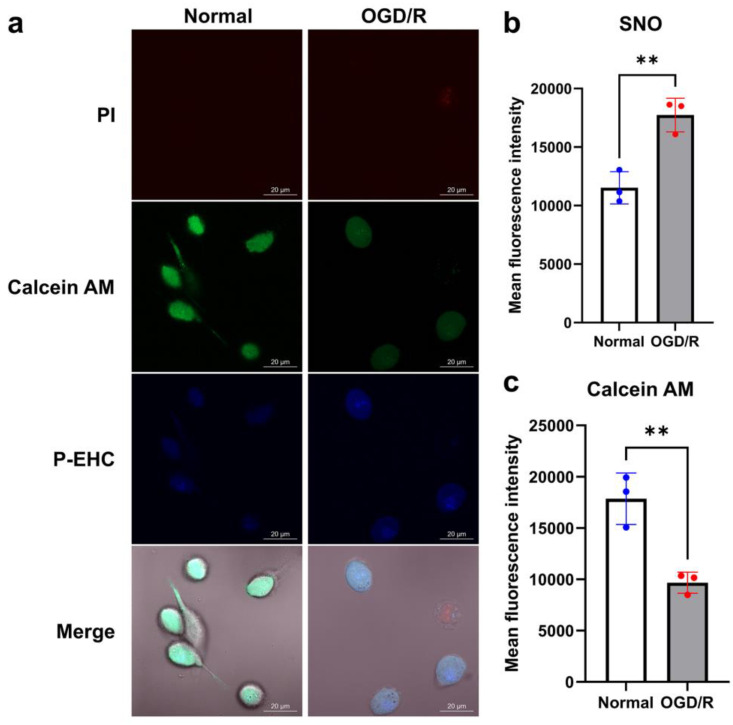
Triple staining for dead cells, live cells, and intracellular SNOs in an OGD/R model. (**a**) Representative confocal micrographs of dead cells (red), live cells (green), and SNOs in SH-SY5Y cells (blue). The merge image represents the overlay of red, green, and blue channels and the bright field. Scale bar = 20 μm. (**b**) Statistical analysis of the **P-EHC** channel shown in (**a**). Each data point represents the mean fluorescence intensity of viable cells from an independent experiment, analyzed by a two-tailed *t*-test. (**c**) Statistical analysis of the Calcein AM channel shown in Figure 5a. Each data point represents the mean fluorescence intensity of viable cells from an independent experiment, analyzed by a two-tailed *t*-test. ** *p* < 0.01. Data are expressed as mean ± SD (*n* = 3).

First, triple staining was performed on OGD/R-treated and untreated SH-SY5Y cells to assess dead cells (PI), live cells (Calcein AM), and SNO levels (**P-EHC**) (Figure 6a). Calcein AM is a nonpolar dye that selectively stains living cells, passively entering and accumulating in them [49]. Once inside, it is hydrolyzed by intracellular esterases, removing methyl acetyl groups and generating fluorescein, which emits strong green fluorescence. PI is a membrane-impermeable dye that binds to DNA, emitting red fluorescence upon excitation. It is commonly used to stain dead or dying cells, as it only penetrates cells with compromised membranes [50].

Confocal imaging revealed significant PI fluorescence in the OGD/R group, indicating increased cell death. Enhanced SNO fluorescence compared to the control group (Figure 6b) indicated elevated SNO levels in OGD/R-induced cells, likely due to increased iNOS expression. The fluorescence from the Calcein AM and **P-EHC** channels showed strong overlap, indicating significant detection of SNOs in live cells. Calcein AM fluorescence was not observed in dead cells, with only faint SNO fluorescence detectable. Additionally, reduced Calcein AM fluorescence intensity suggested lower cell viability in the OGD/R group (Figure 6c).

We further explored the potential mechanisms underlying the increased SNO observed in OGD/R modeling. As described in the Introduction, the conversion of NaNO_2_ [12,13,14] and the upregulation of NOS [9,51] during OGD/R may contribute to intracellular SNO elevation. To validate this, we treated OGD/R-induced SH-SY5Y cells with NaNO_2_ or the NOS inhibitor L-NAME (Figure S10). Confocal imaging and quantitative analysis showed that treatment with 50 μM NaNO_2_ significantly enhanced SNO fluorescence in OGD/R-induced SH-SY5Y cells, highlighting the critical role of hypoxic NaNO_2_ conversion in SNO formation. In contrast, cells treated with 200 μM L-NAME exhibited markedly reduced SNO levels compared to the OGD group, emphasizing the role of NOS as an important source of SNO during OGD/R. Further studies are required to confirm the dose–response relationship.

#### 3.2.2. Protective Effects of **P-EHC** on OGD/R Cells

**Figure 7:**
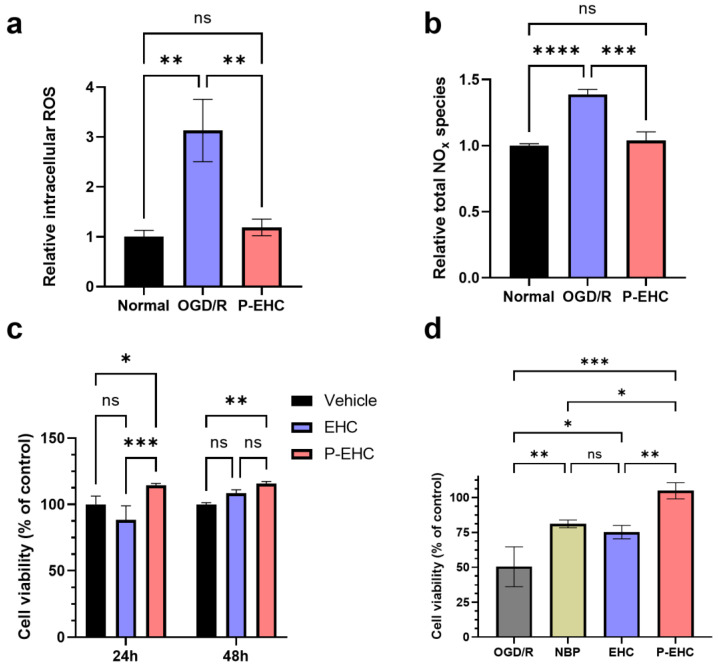
Protective effects of **P-EHC** on OGD/R cells. (**a**) Intracellular ROS levels in SH-SY5Y cells. (**b**) Total NO production in SH-SY5Y cells was determined using a modified Griess assay, which measures NO_x_ species (including endogenous nitrite, nitrite converted from nitrate, and nitrite converted from NO). (**c**) Cell viability of SH-SY5Y cells after treatment with the vehicle, **EHC**, or **P-EHC**, respectively. Analyzed by two-way ANOVA with Šídák’s multiple comparisons test. (**d**) Protective effects of **P-EHC** and **EHC** on OGD/R-induced damage in SH-SY5Y cells. Analyzed by one-way ANOVA with Tukey’s multiple comparisons test. * *p* < 0.05, ** *p* < 0.01, *** *p* < 0.001, **** *p* < 0.0001, ns = non-significant (*p* > 0.05). For (**a**–**d**), all data are presented as the mean ± SD (*n* = 3).

OGD/R-induced overproduction of reactive nitrogen species (RNS) and ROS is a crucial contributor to OGD/R-induced injury [9,52]. In OGD/R-treated SH-SY5Y cells, significant upregulation of intracellular ROS (Figure 7a) and total NO_x_ levels (Figure 7b) was observed, indicating the presence of both nitrosative and oxidative stress. Treatment with **P-EHC** notably reduced ROS (Figure 7a) and total NO_x_ levels (Figure 7b) in OGD/R-treated SH-SY5Y cells (Figure 7a,b). Next, a CCK-8 assay was performed to evaluate the effects of **P-EHC** and **EHC** on cell proliferation at the staining concentration. After 24 h and 48 h of incubation, no significant change in viability was observed in the **EHC** group compared to the vehicle control. Interestingly, the **P-EHC** group showed higher viability, indicating potential proliferative activity (Figure 7c). Finally, we examined the protective effects of **P-EHC** and **EHC** at the staining concentration on OGD/R-induced SH-SY5Y cells, with 5 µM DL-3-n-butylphthalide (NBP) as a positive control. CCK-8 results demonstrated that NBP, **EHC**, and **P-EHC** all conferred protection, with **P-EHC** showing superior efficacy compared to **EHC** (Figure 7d). We propose that **P-EHC**’s ability to scavenge excess SNOs in OGD/R cells is critical to its enhanced protective effect.

## 4. Discussion

To address the poor water solubility of phosphine esters, we designed **EHC**, a water-soluble coumarin derivative that retains MIF inhibitory activity and exhibits a high fluorescence quantum yield. The good water solubility of **EHC** ensures that **P-EHC** possesses adequate solubility in biological systems. **P-EHC** reacts rapidly and selectively with SNOs and enables imaging of SNOs in living cells. In our study, we observed a significant increase in SNO fluorescence using **P-EHC** following OGD/R modeling, which is consistent with previous reports of NO elevation under OGD/R conditions [53,54]. Similarly, a prior study in hepatic ischemia models demonstrated that nitrite administration led to elevated protein SNO levels [55]. In our experiments, treatment of OGD/R cells with 50 μM sodium nitrite further enhanced SNO fluorescence, whereas 200 μM L-NAME suppressed OGD/R-induced SNO fluorescence. These results suggest that the upregulation of SNO levels is likely attributed to the combined effects of NOS-mediated NO production [10,11,56] and the hypoxic conversion pathway of nitrite [12,13,14]. In addition, we observed strong SNO fluorescence in live cells, likely resulting from the continuous synthesis of NO by nitric oxide synthase. In contrast, only faint SNO fluorescence was detected in dead cells, possibly due to the spontaneous breakdown of SNO (Figure 6a).

**P-EHC** reduced total NO_x_ species levels in OGD/R-treated cells. Although **P-EHC** reacts exclusively with SNO, its treatment still resulted in a decrease in total NO_x_, likely because SNO serves as an important storage form for NO [2], and its consumption leads to a reduction in NO_x_ levels. Additionally, we demonstrated that **P-EHC** treatment reduced intracellular ROS levels in OGD/R cells (Figure 7a), likely due to the oxidation of the triphenylphosphine group in **P-EHC** to triphenylphosphine oxide. The reduction in ROS levels may also be linked to hypoxia-inducible factor-1 (HIF-1) activation, which is known to play a central role in cellular adaptation to hypoxic conditions and in modulating the overall cellular response to oxidative stress [57]. Importantly, the oxidation of triphenylphosphine does not cause significant **EHC** release, as evidenced by the reaction with H_2_O_2_ (Figure 5a). Notably, CCK-8 assays demonstrated that **P-EHC** exerts protective effects on SH-SY5Y cells subjected to OGD/R, which we attribute to the synergistic effects of SNO reduction and MIF inhibition.

Despite its potential, **P-EHC** requires improvements in aqueous stability to reduce background fluorescence and enhance the signal-to-noise ratio. A high-fluorescence background is likely a common issue for phosphine ester-based probes, possibly due to the hydrolysis of the ester bond in the probe. As shown in Figure 5b, the change in fluorescence is relatively weak at 50 μM GSNO, indicating that the probe requires a high GSNO concentration to generate a sufficient signal, which is a limitation. To our knowledge, the SNO concentration in cells treated with OGD/R has not yet been reported. For reference, a study reported nearly identical baseline levels of SNOs (1.2 ± 0.4 μM) and NO_2_^−^ (1.3 ± 0.7 μM) in the control group of their AKN-1 cell culture experiment, with significantly elevated levels of SNOs (19.4 ± 6.5 μM) and NO_2_^−^ (61.1 ± 22.3 μM) after cytokine stimulation [58]. The SNO levels in SH-SY5Y cells treated with OGD/R may be in a similar range. While **P-EHC** can effectively detect SNOs in OGDR-treated cells, it may not be suitable for models with lower SNO concentrations. Further optimization of the detection method may be necessary for its application in such models. In addition, ischemia-reperfusion injury involves complex interactions among various cell types, including neurons, endothelial cells, and microglia. Endothelial cells are essential for maintaining the integrity of the blood–brain barrier and regulating cerebral blood flow [59], while microglia, as the primary immune cells in the central nervous system, play a crucial role in modulating inflammatory responses [60]. Our study focuses on the development of the **P-EHC** probe, validating its SNO detection capability and neuroprotective effects. Incorporating these cell types into OGD/R models would provide a more comprehensive understanding of cellular crosstalk and the mechanisms underlying the neuroprotective effects of the compound and ischemia-reperfusion injury. We believe **P-EHC** holds promise for clinical applications, such as monitoring disease progression in pressure ulcers, where its fluorescence intensity correlates with ischemic severity while also promoting wound healing. Future research should focus on developing SNO theranostic probes with enhanced tissue-targeting capabilities, paving the way for novel diagnostic and therapeutic strategies for ischemic diseases, including cerebral and myocardial ischemia.

## 5. Conclusions

In summary, we developed a theranostic SNO probe, **P-EHC**, capable of detecting SNO level changes in cells under OGD/R conditions while simultaneously enhancing cell viability. To our knowledge, this is the first report of such a concept. **P-EHC** serves as a powerful tool for analyzing SNO level dynamics in OGD/R cells and offers new perspectives for the treatment of ischemia-reperfusion diseases.

## Data Availability

Data are contained within the article and Supplementary Materials.

## Supplementary Materials

The following supporting information can be downloaded at: https://www.mdpi.com/article/10.3390/antiox14030311/s1, Figure S1: The NMR spectra of compound **1**; Figure S2: The MS and HRMS spectra of compound **1**; Figure S3: The NMR spectra of **EHC**; Figure S4: The MS and HRMS spectra of **EHC**; Figure S5: The NMR spectra of **P-EHC**; Figure S6: The MS and HRMS spectra of **P-EHC**; Figure S7: The HPLC spectra of **EHC**; Figure S8: The HPLC spectra of **P-EHC**; Figure S9: Relative fluorescence intensity of 100 μM **P-EHC** upon reacting with NONOates; Figure S10: Staining for intracellular SNO in OGD/R-treated SH-SY5Y cells.

## References

[1] Lundberg J.O., Weitzberg E. (2022). Nitric oxide signaling in health and disease. Cell.

[2] Ye H., Wu J., Liang Z., Zhang Y., Huang Z. (2022). Protein S-Nitrosation: Biochemistry, Identification, Molecular Mechanisms, and Therapeutic Applications. J. Med. Chem..

[3] Haldar S.M., Stamler J.S. (2013). S-nitrosylation: Integrator of cardiovascular performance and oxygen delivery. J. Clin. Investig..

[4] Lee H.M., Choi J.W., Choi M.S. (2021). Role of Nitric Oxide and Protein S-Nitrosylation in Ischemia-Reperfusion Injury. Antioxidants.

[5] Lima B., Forrester M.T., Hess D.T., Stamler J.S. (2010). S-nitrosylation in cardiovascular signaling. Circ. Res..

[6] Dasgupta S., Gomez J.J., Singh I., Khan M. (2018). S-Nitrosylation in Regulation of Inflammation and Cell Damage. Curr. Drug Targets.

[7] Liu Z., Ren L., Cui X., Guo L., Jiang B., Zhou J., Liang P., Zeng J., He Z., Zhang P. (2018). Muscular proteomic profiling of deep pressure ulcers reveals myoprotective role of JAK2 in ischemia and reperfusion injury. Am. J. Transl. Res..

[8] Wang Y., Du J., Liu Y., Yang S., Wang Q. (2022). microRNA-301a-3p is a potential biomarker in venous ulcers vein and gets involved in endothelial cell dysfunction. Bioengineered.

[9] Ye X.-C., Hao Q., Ma W.-J., Zhao Q.-C., Wang W.-W., Yin H.-H., Zhang T., Wang M., Zan K., Yang X.-X. (2020). Dectin-1/Syk signaling triggers neuroinflammation after ischemic stroke in mice. J. Neuroinflammation.

[10] Zhang R., Lin Y.Q., Wang W.S., Wang X.Q. (2017). Excessive nNOS/NO/AMPK signaling activation mediated by the blockage of the CBS/H2S system contributes to oxygen-glucose deprivation-induced endoplasmic reticulum stress in PC12 cells. Int. J. Mol. Med..

[11] Wei G., Dawson V.L., Zweier J.L. (1999). Role of neuronal and endothelial nitric oxide synthase in nitric oxide generation in the brain following cerebral ischemia. Biochim. Biophys. Acta Mol. Basis Dis..

[12] Tejero J., Shiva S., Gladwin M.T. (2019). Sources of Vascular Nitric Oxide and Reactive Oxygen Species and Their Regulation. Physiol. Rev..

[13] Monteiro T., Dias C., Lourenço C.F., Ledo A., Barbosa R.M., Almeida M.G. (2021). Microelectrode Sensor for Real-Time Measurements of Nitrite in the Living Brain, in the Presence of Ascorbate. Biosensors.

[14] Du J., Filipović M.R., Wagner B.A., Buettner G.R. (2023). Ascorbate mediates the non-enzymatic reduction of nitrite to nitric oxide. Adv. Redox Res..

[15] Smith B.C., Marletta M.A. (2012). Mechanisms of S-nitrosothiol formation and selectivity in nitric oxide signaling. Curr. Opin. Chem. Biol..

[16] Daiber A., Schildknecht S., Müller J., Kamuf J., Bachschmid M.M., Ullrich V. (2009). Chemical model systems for cellular nitros(yl)ation reactions. Free Radic. Biol. Med..

[17] Schildknecht S., von Kriegsheim A., Vujacic-Mirski K., Di Lisa F., Ullrich V., Daiber A. (2022). Recovery of reduced thiol groups by superoxide-mediated denitrosation of nitrosothiols. Redox Biol..

[18] Yoon S., Eom G.H., Kang G. (2021). Nitrosative Stress and Human Disease: Therapeutic Potential of Denitrosylation. Int. J. Mol. Sci..

[19] Sun J., Murphy E. (2010). Protein S-nitrosylation and cardioprotection. Circ. Res..

[20] Liu C., Park C.-M., Wang D., Xian M. (2018). Phosphite Esters: Reagents for Exploring S-Nitrosothiol Chemistry. Org. Lett..

[21] Zhang D., Devarie-Baez N.O., Pan J., Wang H., Xian M. (2010). One-Pot Thioether Formation from S-Nitrosothiols. Org. Lett..

[22] Zhang C., Biggs T.D., Devarie-Baez N.O., Shuang S., Dong C., Xian M. (2017). S-Nitrosothiols: Chemistry and reactions. Chem. Commun..

[23] Zhang J., Li S., Zhang D., Wang H., Whorton A.R., Xian M. (2010). Reductive ligation mediated one-step disulfide formation of S-nitrosothiols. Org. Lett..

[24] Pan J., Downing J.A., McHale J.L., Xian M. (2009). A fluorogenic dye activated by S-nitrosothiols. Mol. Biosyst..

[25] Zhang D., Chen W., Miao Z., Ye Y., Zhao Y., King S.B., Xian M. (2014). A reductive ligation based fluorescent probe for S-nitrosothiols. Chem. Commun..

[26] Shao S., Chen B., Cheng J., Wang C., Zhang Y., Shao L., Hu Y., Han Y., Han F., Li X. (2017). A fluorogenic probe for imaging protein S-nitrosylation in live cells. Biosens. Bioelectron..

[27] Yu J., Gan L., Zhou Y., Xu J., Yun C., Fang T., Cai X. (2022). Indole-Based Long-Wavelength Fluorescent Probes for Bioimaging of S-Nitrosylation in Mitochondria. Chem. Eur. J..

[28] Wang H., Xian M. (2008). Fast reductive ligation of S-nitrosothiols. Angew. Chem. Int. Ed..

[29] Seneviratne U., Godoy L.C., Wishnok J.S., Wogan G.N., Tannenbaum S.R. (2013). Mechanism-based triarylphosphine-ester probes for capture of endogenous RSNOs. J. Am. Chem. Soc..

[30] García-Arellano S., Hernández-Palma L.A., Cerpa-Cruz S., Sánchez-Zuno G.A., Herrera-Godina M.G., Muñoz-Valle J.F. (2021). The Novel Role of MIF in the Secretion of IL-25, IL-31, and IL-33 from PBMC of Patients with Rheumatoid Arthritis. Molecules.

[31] Caltabiano R., De Pasquale R., Piombino E., Campo G., Nicoletti F., Cavalli E., Mangano K., Fagone P. (2021). Macrophage Migration Inhibitory Factor (MIF) and Its Homologue D-Dopachrome Tautomerase (DDT) Inversely Correlate with Inflammation in Discoid Lupus Erythematosus. Molecules.

[32] Petralia M.C., Battaglia G., Bruno V., Pennisi M., Mangano K., Lombardo S.D., Fagone P., Cavalli E., Saraceno A., Nicoletti F. (2020). The Role of Macrophage Migration Inhibitory Factor in Alzheimer′s Disease: Conventionally Pathogenetic or Unconventionally Protective?. Molecules.

[33] Vámos E., Kálmán N., Sturm E.M., Nayak B.B., Teppan J., Vántus V.B., Kovács D., Makszin L., Loránd T., Gallyas F. (2023). Highly Selective MIF Ketonase Inhibitor KRP-6 Diminishes M1 Macrophage Polarization and Metabolic Reprogramming. Antioxidants.

[34] Li W.H., Yang Y.L., Cheng X., Liu M., Zhang S.S., Wang Y.H., Du G.H. (2020). Baicalein attenuates caspase-independent cells death via inhibiting PARP-1 activation and AIF nuclear translocation in cerebral ischemia/reperfusion rats. Apoptosis.

[35] Wang L., Zis O., Ma G., Shan Z., Zhang X., Wang S., Dai C., Zhao J., Lin Q., Lin S. (2009). Upregulation of macrophage migration inhibitory factor gene expression in stroke. Stroke.

[36] Zis O., Zhang S., Dorovini-Zis K., Wang L., Song W. (2015). Hypoxia signaling regulates macrophage migration inhibitory factor (MIF) expression in stroke. Mol. Neurobiol..

[37] Liang C.J., Li J.H., Zhang Z., Zhang J.Y., Liu S.Q., Yang J. (2018). Suppression of MIF-induced neuronal apoptosis may underlie the therapeutic effects of effective components of Fufang Danshen in the treatment of Alzheimer’s disease. Acta Pharmacol. Sin..

[38] Li Y., Zou C., Chen C., Li S., Zhu Z., Fan Q., Pang R., Li F., Chen Z., Wang Z. (2023). Myeloid-derived MIF drives RIPK1-mediated cerebromicrovascular endothelial cell death to exacerbate ischemic brain injury. Proc. Natl. Acad. Sci. USA.

[39] Liu Y.C., Tsai Y.H., Tang S.C., Liou H.C., Kang K.H., Liou H.H., Jeng J.S., Fu W.M. (2018). Cytokine MIF Enhances Blood-Brain Barrier Permeability: Impact for Therapy in Ischemic Stroke. Sci. Rep..

[40] Meyer-Siegler K.L., Iczkowski K.A., Leng L., Bucala R., Vera P.L. (2006). Inhibition of Macrophage Migration Inhibitory Factor or Its Receptor (CD74) Attenuates Growth and Invasion of DU-145 Prostate Cancer Cells1. J. Immunol..

[41] Wen J., Zhu L.L., Bi Q.W., Shen Z.Q., Li X.X., Li X., Wang Z., Chen Z. (2014). Highly N2-selective coupling of 1,2,3-triazoles with indole and pyrrole. Chemistry.

[42] Sadhu K.K., Mizukami S., Yoshimura A., Kikuchi K. (2013). pH induced dual “OFF-ON-OFF” switch: Influence of a suitably placed carboxylic acid. Org. Biomol. Chem..

[43] Molnar V., Garai J. (2005). Plant-derived anti-inflammatory compounds affect MIF tautomerase activity. Int. Immunopharmacol..

[44] Orita M., Yamamoto S., Katayama N., Aoki M., Takayama K., Yamagiwa Y., Seki N., Suzuki H., Kurihara H., Sakashita H. (2001). Coumarin and chromen-4-one analogues as tautomerase inhibitors of macrophage migration inhibitory factor: Discovery and X-ray crystallography. J. Med. Chem..

[45] McLean L.R., Zhang Y., Li H., Choi Y.M., Han Z., Vaz R.J., Li Y. (2010). Fragment screening of inhibitors for MIF tautomerase reveals a cryptic surface binding site. Bioorg. Med. Chem. Lett..

[46] Kočí J., Grandclaude V., Massonneau M., Richard J.A., Romieu A., Renard P.Y. (2011). A novel and unusually long-lived chemiluminophore based on the 7-hydroxycoumarin scaffold. Chem. Commun..

[47] Rizza S., Montagna C., Cardaci S., Maiani E., Di Giacomo G., Sanchez-Quiles V., Blagoev B., Rasola A., De Zio D., Stamler J.S. (2016). S-nitrosylation of the Mitochondrial Chaperone TRAP1 Sensitizes Hepatocellular Carcinoma Cells to Inhibitors of Succinate Dehydrogenase. Cancer Res..

[48] Lakshmi V.M., Hsu F.F., Zenser T.V. (2005). Nitric oxide-mediated nitrosation of 2-amino-3,8-dimethylimidazo[4,5-f]quinoxaline potentiated by hemin and myeloperoxidase. Chem. Res. Toxicol..

[49] Yu M., Jiang Y., Feng Q., Ouyang Y., Gan J. (2014). DRAM1 protects neuroblastoma cells from oxygen-glucose deprivation/reperfusion-induced injury via autophagy. Int. J. Mol. Sci..

[50] Riccardi C., Nicoletti I. (2006). Analysis of apoptosis by propidium iodide staining and flow cytometry. Nat. Protoc..

[51] Guo B., Ma B., Li M., Li Y., Liang P., Han D., Yan X., Hu S. (2024). The nitration of SIRT6 aggravates neuronal damage during cerebral ischemia-reperfusion in rat. Nitric Oxide.

[52] Pérez-Torres I., Manzano-Pech L., Rubio-Ruíz M.E., Soto M.E., Guarner-Lans V. (2020). Nitrosative Stress and Its Association with Cardiometabolic Disorders. Molecules.

[53] Lee D.-H., Lee Y.J., Kwon K.H. (2010). Neuroprotective Effects of Astaxanthin in Oxygen-Glucose Deprivation in SH-SY5Y Cells and Global Cerebral Ischemia in Rat. J. Clin. Biochem. Nutr..

[54] Hsieh Y.S., Shin Y.K., Seol G.H. (2021). Protection of the neurovascular unit from calcium-related ischemic injury by linalyl acetate. Chin. J. Physiol..

[55] Duranski M.R., Greer J.J.M., Dejam A., Jaganmohan S., Hogg N., Langston W., Patel R.P., Yet S.-F., Wang X., Kevil C.G. (2005). Cytoprotective effects of nitrite during in vivo ischemia-reperfusion of the heart and liver. J. Clin. Investig..

[56] Xu J., He L., Ahmed S.H., Chen S.-W., Goldberg M.P., Beckman J.S., Hsu C.Y. (2000). Oxygen-Glucose Deprivation Induces Inducible Nitric Oxide Synthase and Nitrotyrosine Expression in Cerebral Endothelial Cells. Stroke.

[57] Semenza G.L. (2014). Hypoxia-inducible factor 1 and cardiovascular disease. Annu. Rev. Physiol..

[58] Marzinzig M., Nussler A.K., Stadler J., Marzinzig E., Barthlen W., Nussler N.C., Beger H.G., Morris S.M., Brückner U.B. (1997). Improved methods to measure end products of nitric oxide in biological fluids: Nitrite, nitrate, and S-nitrosothiols. Nitric Oxide.

[59] Jakubowski H., Witucki Ł. (2025). Homocysteine Metabolites, Endothelial Dysfunction, and Cardiovascular Disease. Int. J. Mol. Sci..

[60] Qin C., Zhou L.Q., Ma X.T., Hu Z.W., Yang S., Chen M., Bosco D.B., Wu L.J., Tian D.S. (2019). Dual Functions of Microglia in Ischemic Stroke. Neurosci. Bull..

