# Supplementary material for: A Fluorescent Probe for Imaging and Treating S-Nitrosation Stress in OGD/R Cells
Source: Antioxidants (Basel). 2025 Mar 4;14(3):311. doi: 10.3390/antiox14030311 (PMC11939710; doi:10.3390/antiox14030311)
Supplement: Supplementary file 1 [file antioxidants-14-00311-s001.zip › antioxidants-3452609-supplementary.pdf]

Supporting information for

A Fluorescent Probe for Imaging and Treating of S-Nitrosation Stress in OGD/R Cells

Hui Ye [†,1], Chen Zhang [†,1], Lerong Li [1], Cunrui Li [1], Jiayue Yu [1], Duorui Ji [1], Zhuangzhuang Liang [1], Jianbing Wu [*,1], Zhangjian Huang [*,1,2]

* Jianbing Wu − State Key Laboratory of Natural Medicines, China Pharmaceutical University, Nanjing 211198, China;
orcid.org/0000-0003-2725-9859;
Email: jwu@cpu.edu.cn

* Zhangjian Huang − State Key Laboratory of Natural Medicines, China Pharmaceutical University, Nanjing 211198, China; School of Pharmacy, Xinjiang Key Laboratory of Biopharmaceuticals and Medical Devices, Key Laboratory of Active Components of Xinjiang Natural Medicine and Drug Release Technology, Engineering Research Center of Xinjiang and Central Asian Medicine Resources, Xinjiang Medical University, Urumqi 830054, China.
Email: zhangjianhuang@cpu.edu.cn

† These authors contributed equally to this work.

Table of contents

^{1}H NMR, ^{13}C NMR, MS and HRMS Spectra……S2

HPLC Spectra for Target Compounds……S8

Supplementary Figures……S9

^{1}H NMR, ^{13}C NMR, MS, and HRMS Spectra

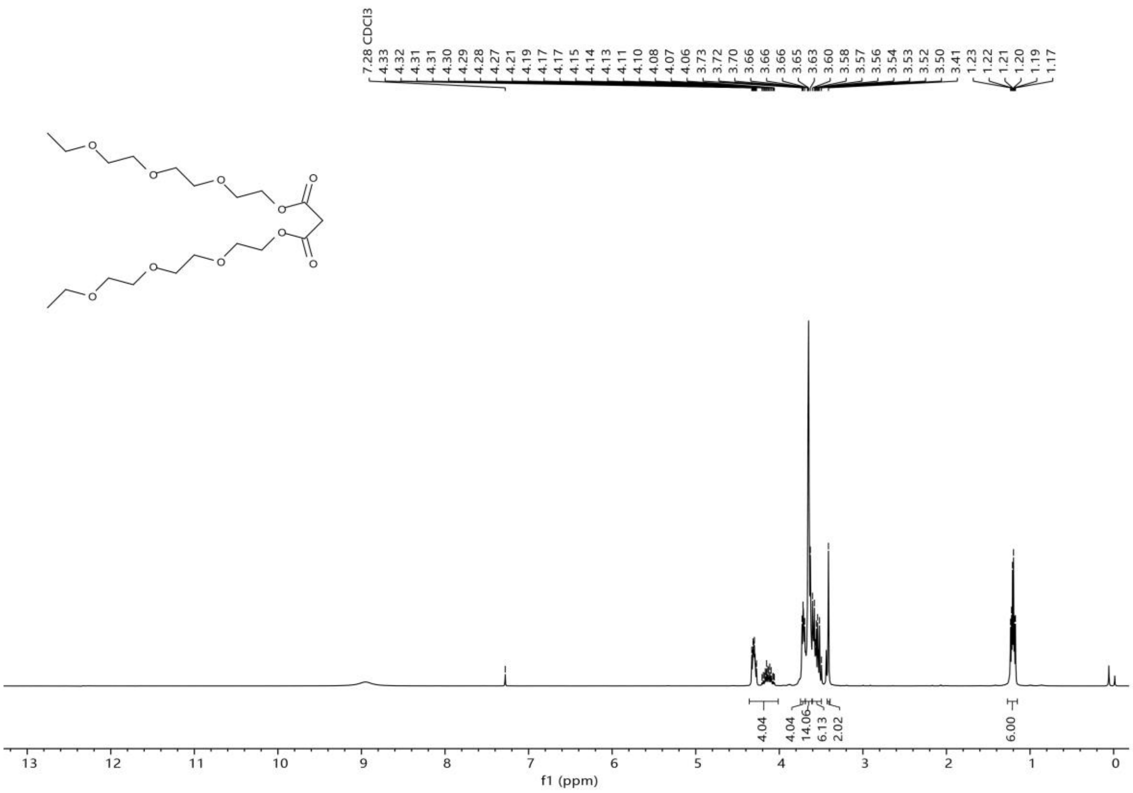

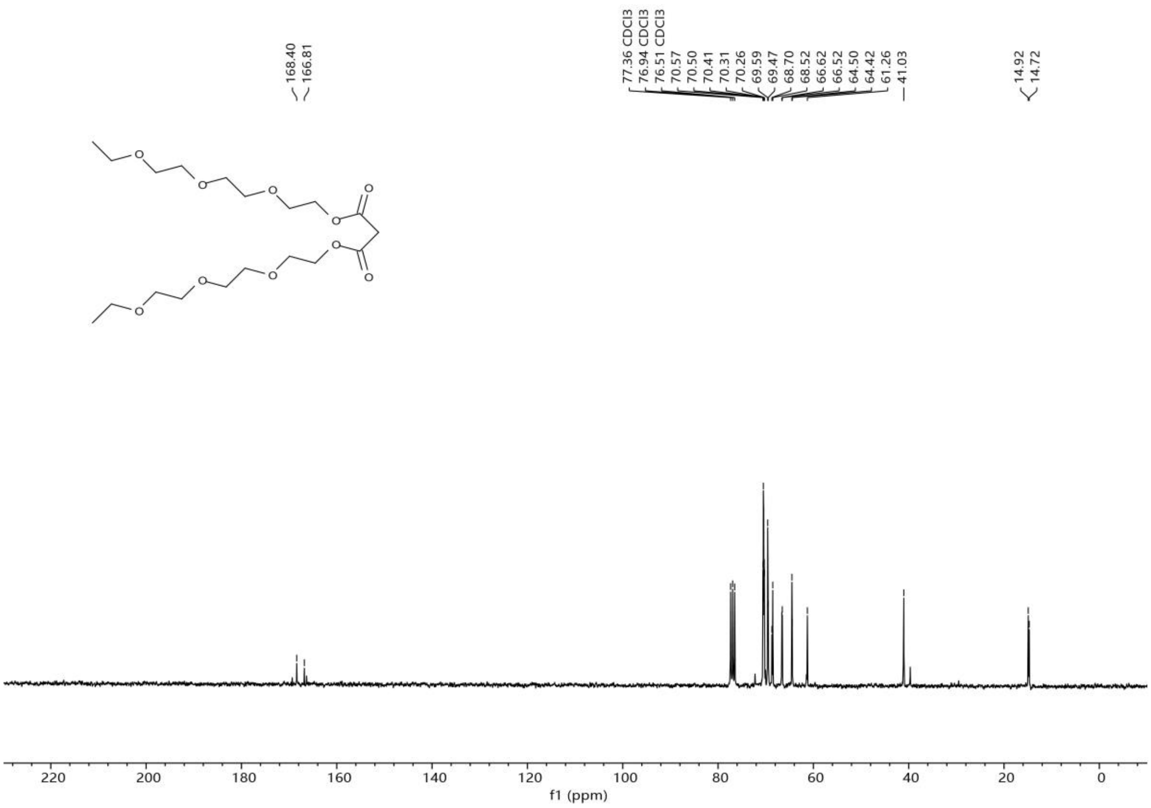

Figure S1. The NMR spectra of compound **1**.

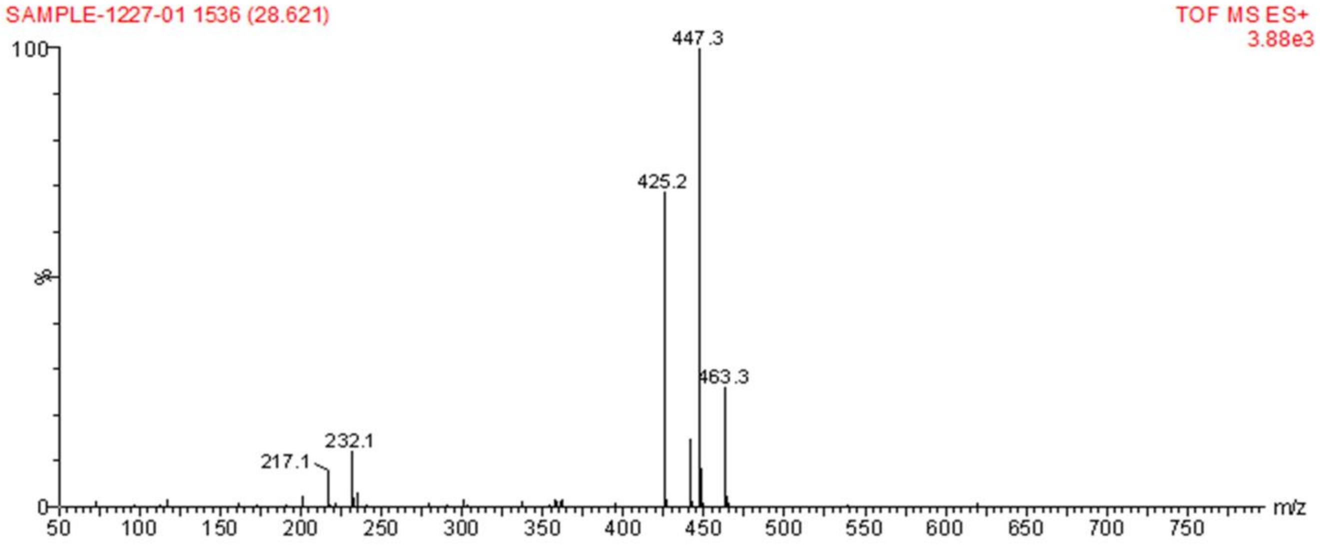

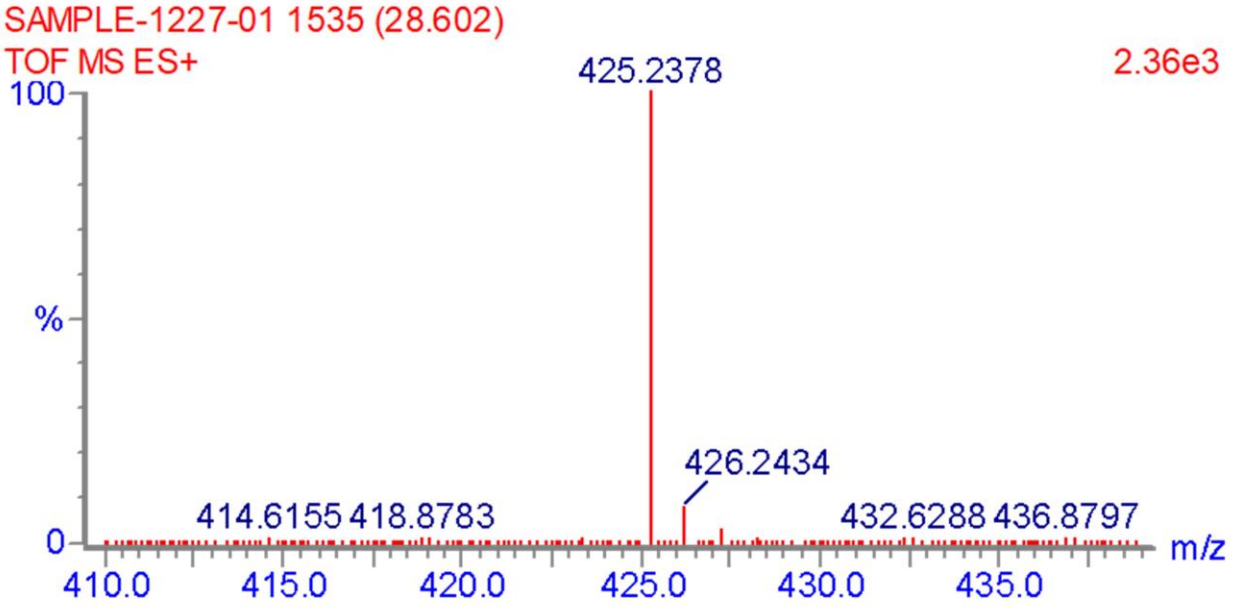

Figure S2. The MS and HRMS spectra of compound **1**.

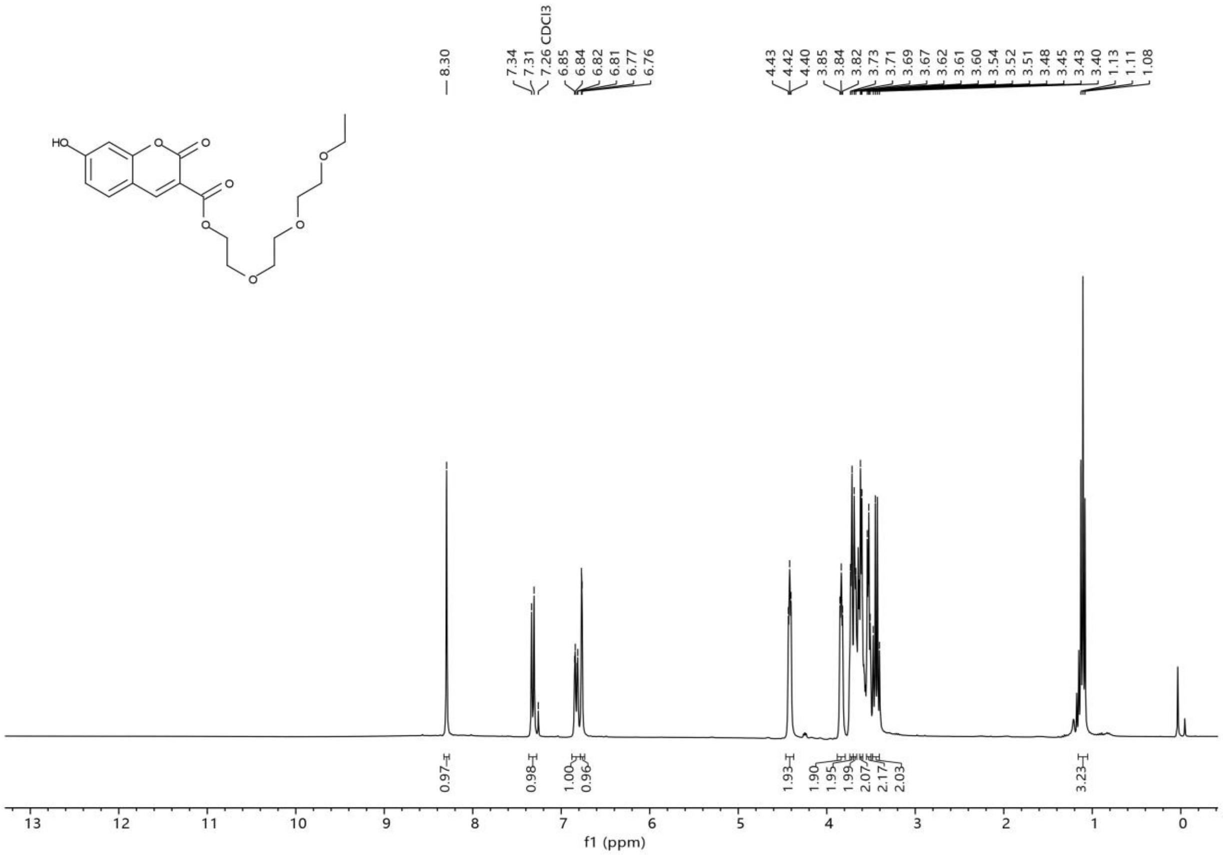

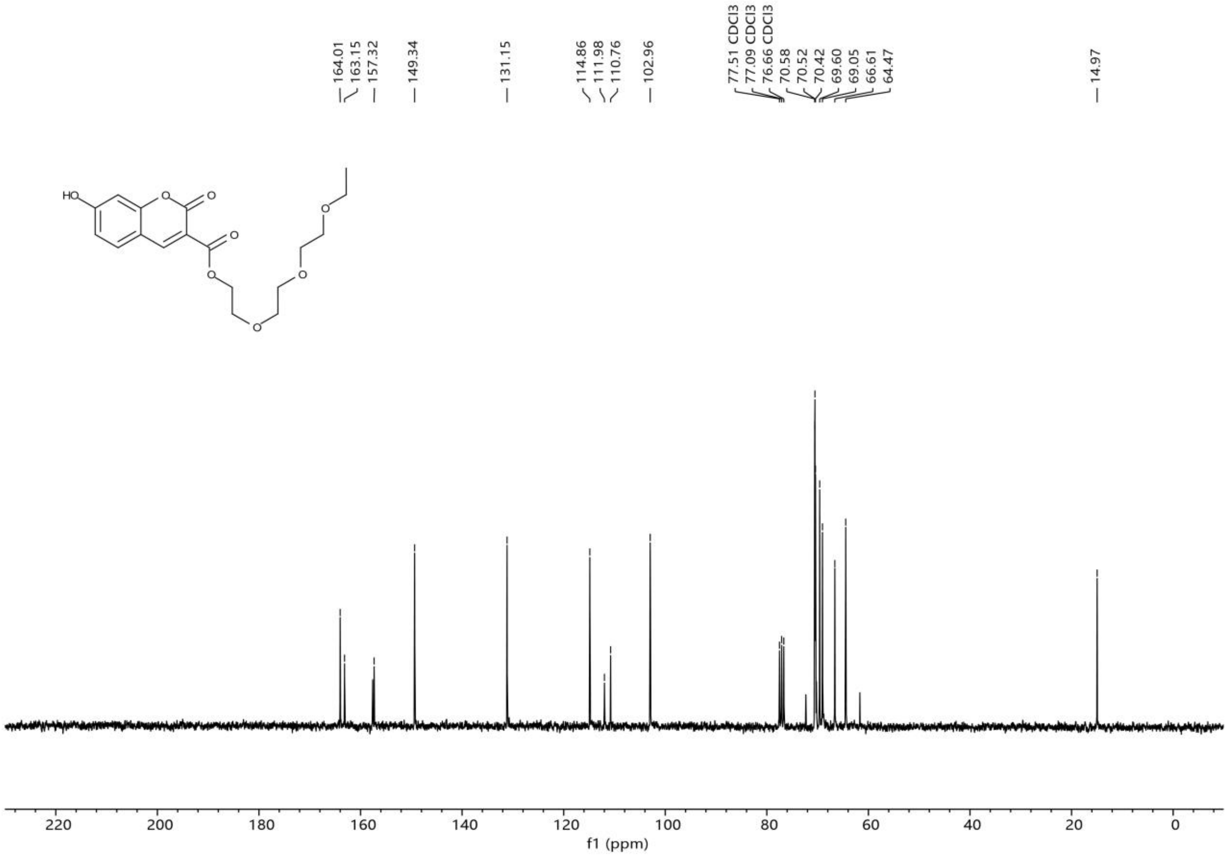

Figure S3. The NMR spectra of **EHC**.

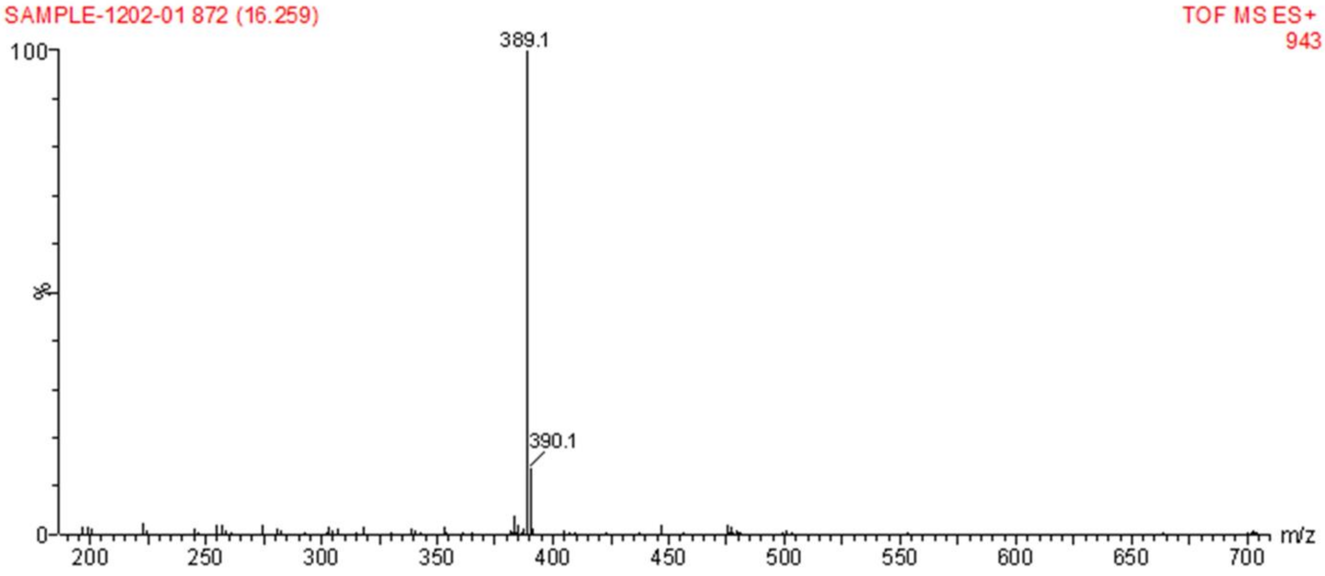

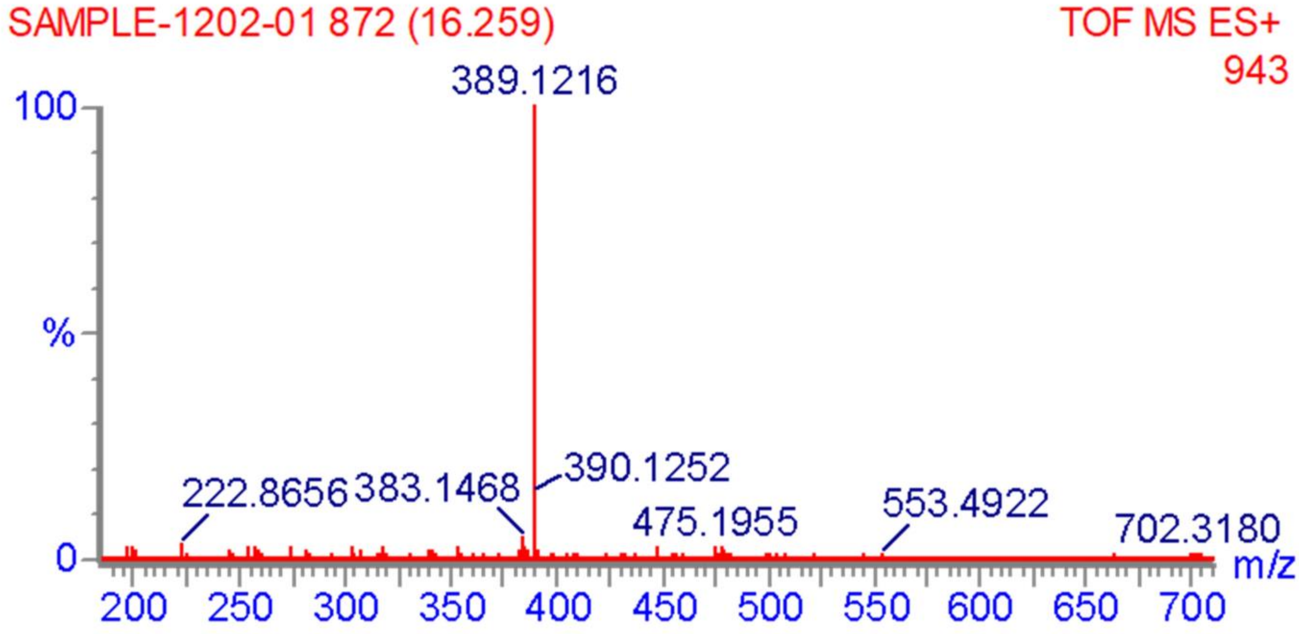

Figure S4. The MS and HRMS spectra of **EHC**.

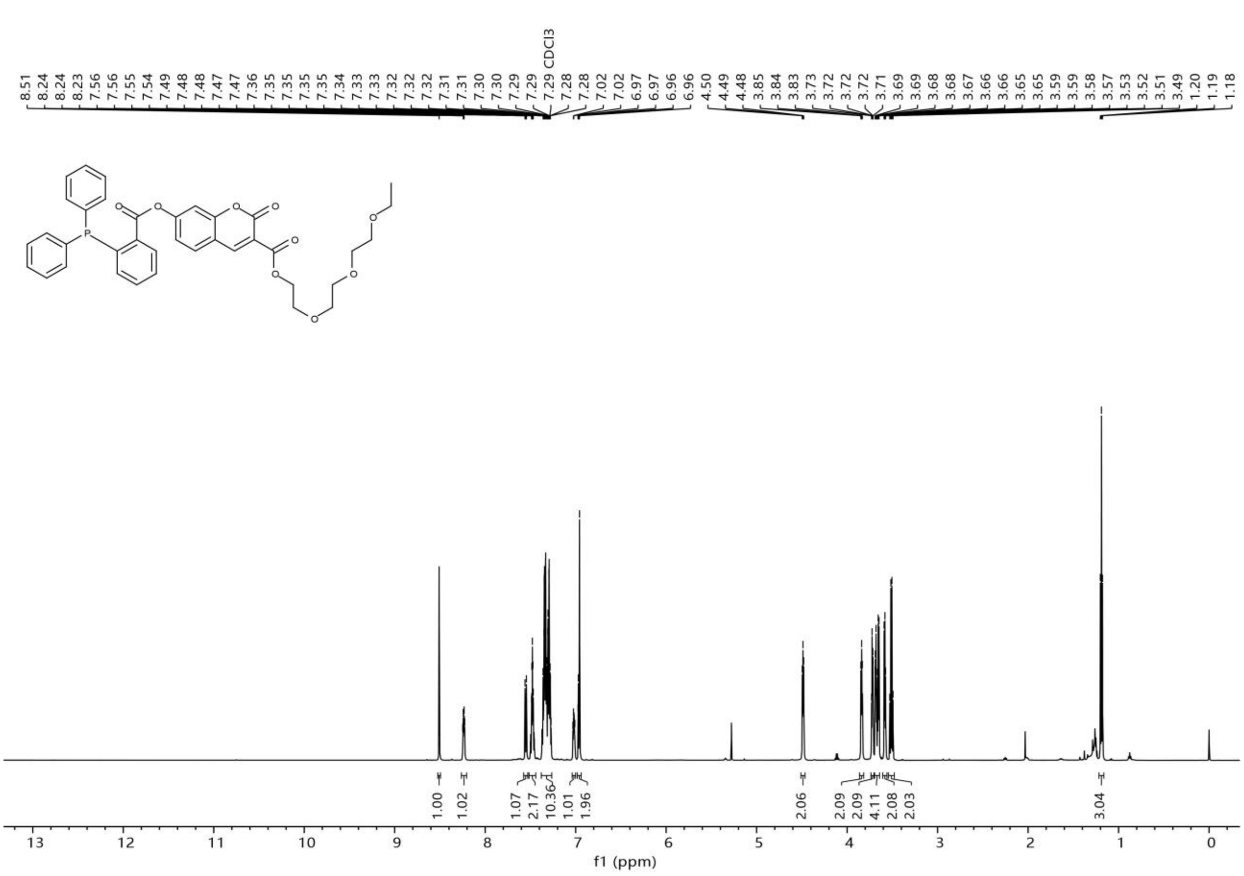

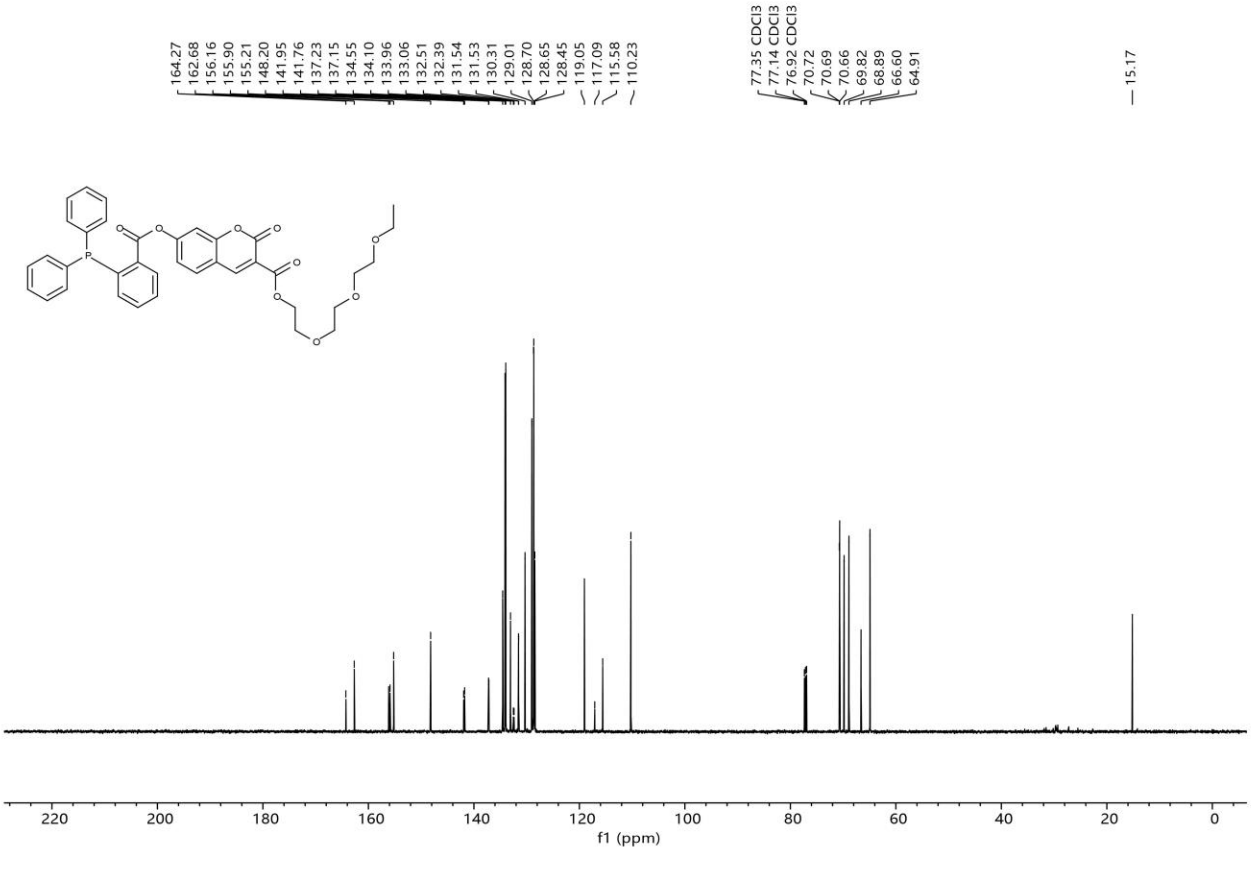

Figure S5. The NMR spectra of **P-EHC**.

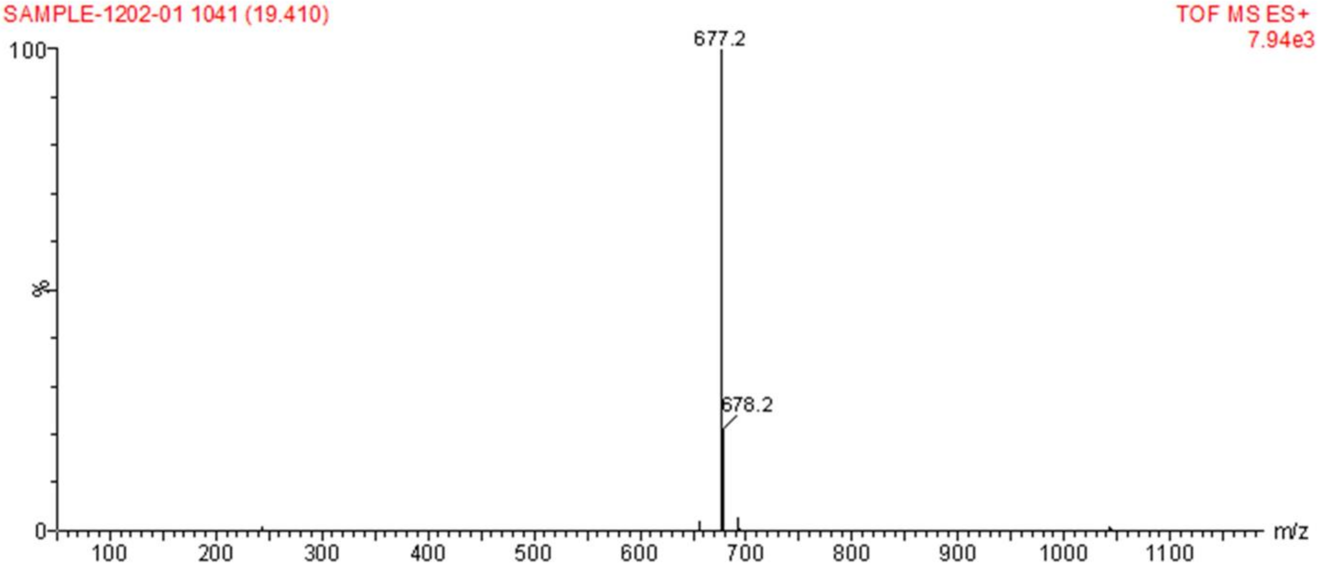

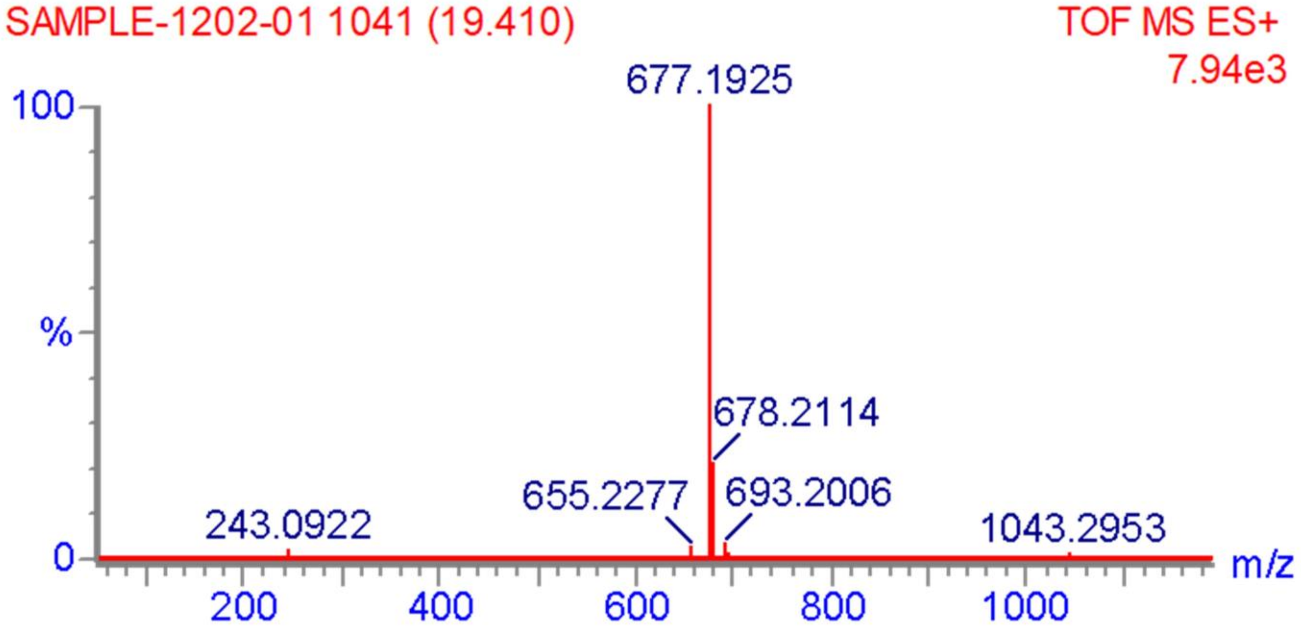

Figure S6. The MS and HRMS spectra of **P-EHC**.

HPLC Spectra for Target Compounds

All tested compounds (**EHC**, **P-EHC**) with a purity of > 95% were used for subsequent biological assays. We provided the spectra of HPLC assays as below. Column: Welch Ultimate XB-C18 (250 mm × 4.6 mm × 5 μm); Detector: UV detector Wavelength: 254 nm; Temperature: 25 ℃. For **EHC**, purity: 97.01%; mobile phase: MeOH:H_2O=7:3; flow rate: 0.5 mL/min. For **P-EHC**, purity: 97.50%; mobile phase: MeOH:H_2O=3:1; flow rate: 1.0 mL/min.

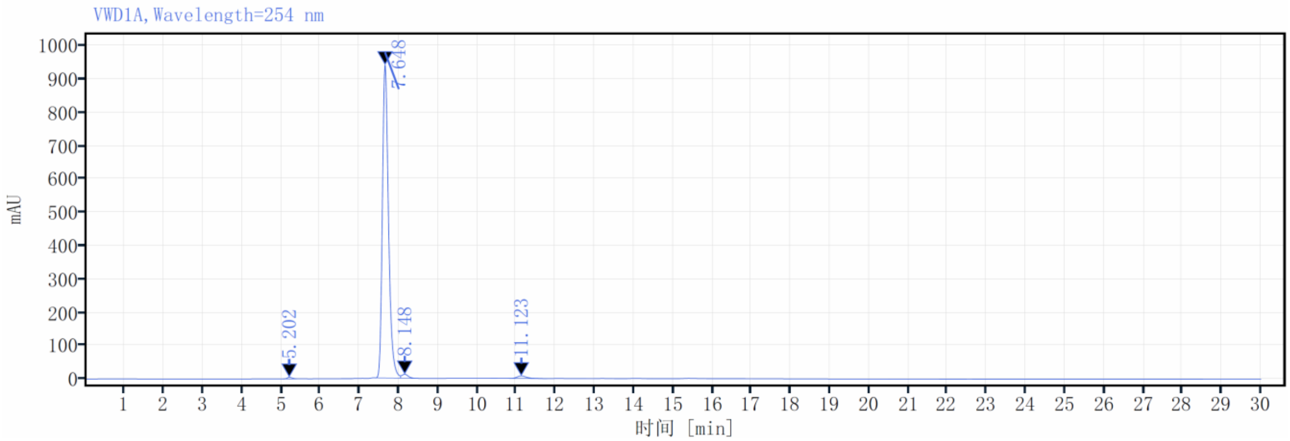

Figure S7. The HPLC spectra of **EHC**.

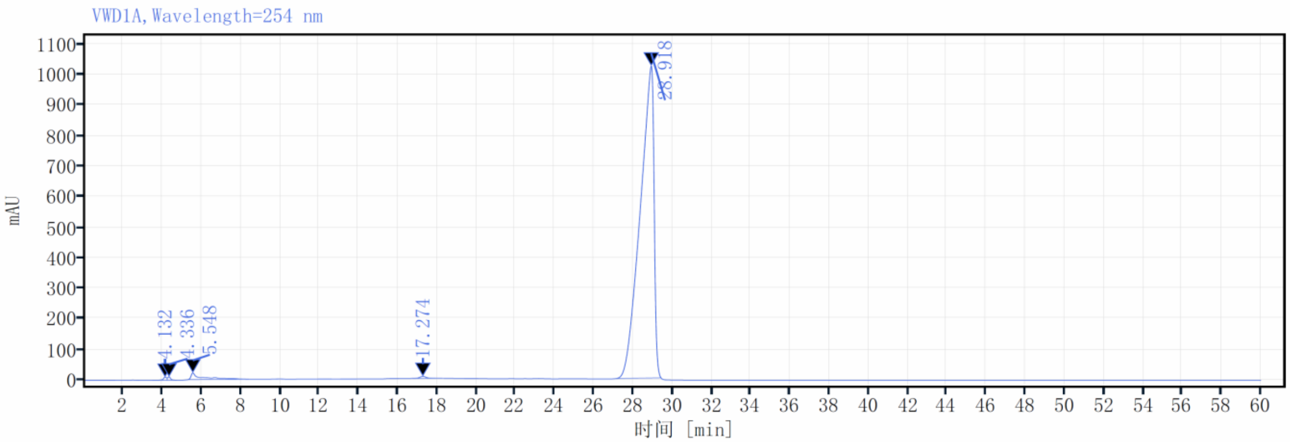

Figure S8. The HPLC spectra of **P-EHC**.

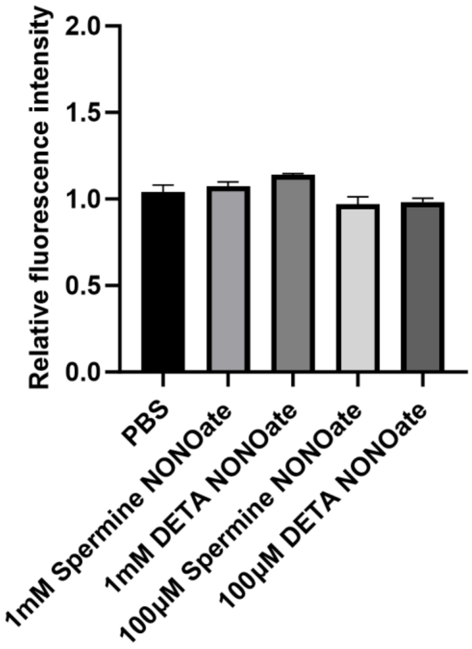

Figure S9. Relative fluorescence intensity of 100 μM **P-EHC** upon reaction with NONOates. **P-EHC** was dissolved in PBS containing 5% DMSO and mixed with an equal volume of reactant. The mixture was shaken at room temperature for 15min, and fluorescence was measured using a microplate reader (Berthold Tristar5). λ_{ex} = 402 nm, λ_{em} = 438nm.

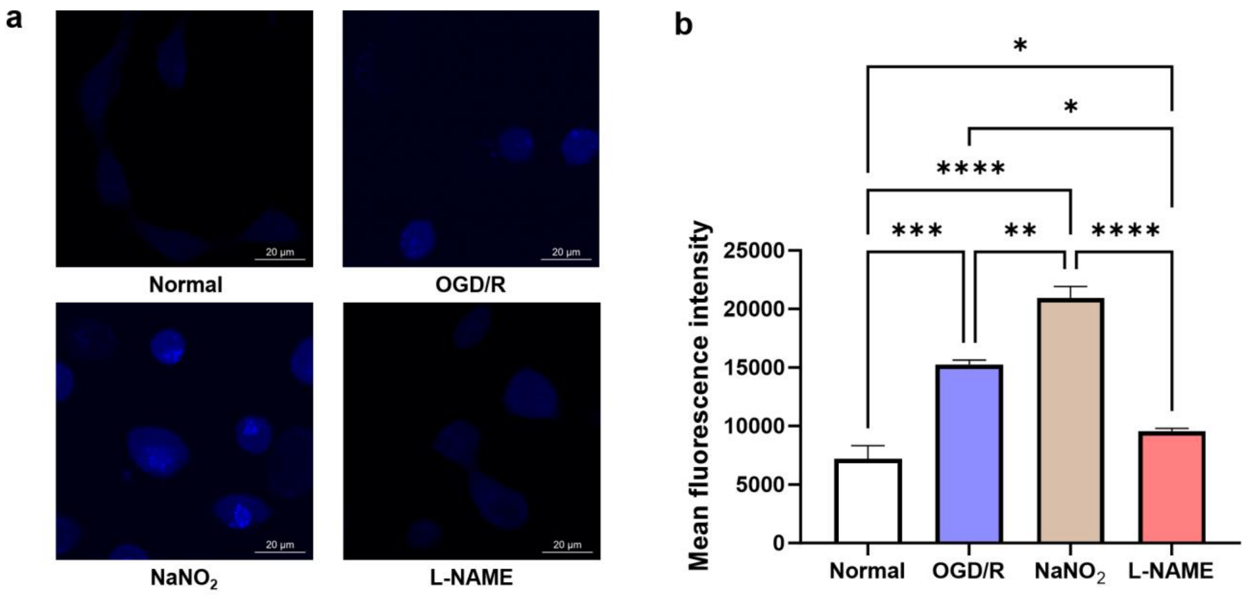

Figure S10. Staining for intracellular SNOs in OGD/R-treated SH-SY5Y cells. (**a**) Representative confocal micrographs of SNOs in normal cells, OGD/R cells, $NaNO_2$ (50 μM) treated ODG/R cells and L-NAME (200 μM) treated ODG/R cells. Scale bar = 20 μm. (**b**) Statistical analysis of **P-EHC** channel shown in (**a**). Analyzed by one-way ANOVA with Tukey's multiple comparisons test. *P < 0.05, **P < 0.01, ***P < 0.001, ****P < 0.0001, ns = non-significant (P > 0.05). Data are expressed as mean ± SD (n = 3).
